# Glucose control and psychosocial outcomes with use of automated insulin delivery for 12 to 96 weeks in type 1 diabetes: a meta-analysis of randomised controlled trials

**DOI:** 10.1186/s13098-023-01144-4

**Published:** 2023-09-28

**Authors:** Amanda Godoi, Isabela Reis Marques, Eduardo M. H. Padrão, Ashwin Mahesh, Larissa C. Hespanhol, José Eduardo Riceto Loyola Júnior, Isabela A. F. de Souza, Vittoria C. S. Moreira, Caroliny H. Silva, Isabele A. Miyawaki, Christi Oommen, Cintia Gomes, Ariadne C. Silva, Kavita Advani, Joao Roberto de Sa

**Affiliations:** 1 Cardiff University School of Medicine, Neuadd Meirionnydd, Cardiff, CF144YS UK; 2 Universitat Internacional de Catalunya, Barcelona, Spain; 3 Massachusetts General Hospital, Harvard Medical School, Boston, MA USA; 4 University of Connecticut Health, Farmington, USA; 5 Federal University of Campina Grande, Cajazeiras, Brazil; 6 Hospital Alemão Oswaldo Cruz, São Paulo, Brazil; 7 Petrópolis Medical School, Petrópolis, Brazil; 8 University Israelita de Ciências da Saúde Albert Einstein, São Paulo, Brazil; 9 Federal University of Rio Grande do Norte, Natal, Brazil; 10 Federal University of Paraná, Curitiba, Brazil; 11 Federal University of Santa Maria, Santa Maria, Brazil; 12 UniEvangelica University Centre of Anapolis, Anapolis, Brazil; 13 Endocrinology Division, ABC School of Medicine and Federal University of Sao Paulo, Paulista School of Medicine, São Paulo, Brazil

**Keywords:** Closed-loop, Automated insulin delivery, HbA1c, TIR, Time in range, Hypoglycaemia, Glucose control, Diabetes technology, Type 1 diabetes, T1DM

## Abstract

**Background:**

Glycaemic control of Type 1 Diabetes Mellitus (T1DM) remains a challenge due to hypoglycaemic episodes and the burden of insulin self-management. Advancements have been made with the development of automated insulin delivery (AID) devices, yet, previous reviews have only assessed the use of AID over days or weeks, and potential benefits with longer time of AID use in this population remain unclear.

**Methods:**

We performed a systematic review and meta-analysis of randomised controlled trials comparing AID (hybrid and fully closed-loop systems) to usual care (sensor augmented pumps, multiple daily insulin injections, continuous glucose monitoring and predictive low-glucose suspend) for adults and children with T1DM with a minimum duration of 3 months. We searched PubMed, Embase, Cochrane Central, and Clinicaltrials.gov for studies published up until April 4, 2023. Main outcomes included time in range 70–180 mg/dL as the primary outcome, and change in HbA1c (%, mmol/mol), glucose variability, and psychosocial impact (diabetes distress, treatment satisfaction and fear of hypoglycaemia) as secondary outcomes. Adverse events included diabetic ketoacidosis (DKA) and severe hypoglycaemia. Statistical analyses were conducted using mean differences and odds ratios. Sensitivity analyses were performed according to age, study duration and type of AID device. The protocol was registered in PROSPERO, CRD42022366710.

**Results:**

We identified 25 comparisons from 22 studies (six crossover and 16 parallel designs) including a total of 2376 participants (721 in adult studies, 621 in paediatric studies, and 1034 in combined studies) which were eligible for analysis. Use of AID devices ranged from 12 to 96 weeks. Patients using AID had 10.87% higher time in range [95% CI 9.38 to 12.37; p < 0.0001, I^2^ = 87%) and 0.37% (4.77 mmol/mol) lower HbA1c (95% CI − 0.49% (− 6.39 mmol/mol) to – 0.26 (− 3.14 mmol/mol); p < 0·0001, I^2^ = 77%]. AID systems decreased night hypoglycaemia, time in hypoglycaemia and hyperglycaemia and improved patient distress, with no increase in the risk of DKA or severe hypoglycaemia. No difference was found regarding treatment satisfaction or fear of hypoglycaemia. Among children, there was no difference in glucose variability or time spent in hypoglycaemia between the use of AID systems or usual care. In sensitivity analyses, results remained consistent with the overall analysis favouring AID.

**Conclusion:**

The use of AID systems over 12 weeks, regardless of technical or clinical differences, improved glycaemic outcomes and diabetes distress without increasing the risk of adverse events in adults and children with T1DM.

**Supplementary Information:**

The online version contains supplementary material available at 10.1186/s13098-023-01144-4.

## Background

Type 1 diabetes mellitus (T1DM) is a chronic autoimmune disease, characterised by the progressive destruction of pancreatic beta cells [1, 2]. Intensive insulin treatment is the current standard of care for T1DM. Unfortunately, the proportion of patients achieving a controlled HbA1c and their time in range (TIR) glycaemic level is low. A large proportion of individuals with type 1 diabetes are unable to meet recommended glycaemic targets [3, 4] and severe hypoglycaemia is a recurrent problem [5].

Since the 1960s, several automated insulin delivery (AID) systems have been developed. The goal of such devices is to achieve better glycaemic control, reduce glucose variability, and decrease the risk of micro and macrovascular complications as well as treatment distress [6]. An AID system consists of three components: a continuous glucose monitor (CGM), a pump able to continuously deliver insulin, and a computer algorithm controlling insulin delivery through glucose-responsive feedback [7]. In the last 15 years, multiple closed-loop (CL) systems were developed, such as predictive low-glucose suspend (PLGS) systems, hybrid closed-loop (HCL) systems, and fully closed-loop (FCL) systems, however, their long-term impact on clinical and functional outcomes is still unclear. Previous randomised controlled trials (RCTs) have obtained variable conclusions. While some showed no significant difference in mean overnight blood glucose when comparing CL and Sensor-augmented Insulin Pump (SAP) in adults [8], adolescents [9], and children [10], others showed no difference in time spent in hypoglycaemia [11]. Recent trials using more advanced AID systems have demonstrated better therapeutic efficacy regarding HbA1c levels and TIR [12].

During the last decade, several meta-analyses of RCTs have been reported and show encouraging results on the effectiveness of AID devices in optimising glycaemic control, but assessments have only focused on studies with limited time of AID use, mostly hours or days [13]. To our knowledge, only one published meta-analysis with 11 RCTs has discussed the potential of these devices up to 8 weeks of use [14]. However, no previous meta-analysis has exclusively assessed studies with over 12 weeks of AID use, which is a more appropriate period of time to properly detect changes in HbA1c levels [15]. Furthermore, we did not find any meta-analyses assessing the longer use of AID systems according to different age groups compared to usual care (UC), which currently represents the use of multiple daily insulin injections (MDII), SAP, CGM or PLGS. Lastly, severe adverse events (AEs) and psychosocial outcomes, which can influence clinical decisions, have not yet been assessed in the setting of longer and continuous use of AID systems.

In this updated systematic review and meta-analysis, our objective was to investigate the impact of AID systems compared to UC on glucose control, as well as treatment satisfaction and distress based on the evidence from RCTs with a duration above 12 weeks. We aimed to determine whether the use of AID systems improved TIR, HbA1c, and glycaemic variability, reduced AEs, and impacted psychosocial outcomes from a functional perspective.

## Methods

This review was performed in line with the Preferred Reporting Items for Systematic Reviews and Meta-Analysis (PRISMA) Statement and recommendations of the Cochrane Collaboration Handbook for Systematic Reviews of Interventions [16]. The protocol of this meta-analysis was registered on PROSPERO on October 22, 2022 (ID CRD42022366710).

### Search strategy

We systematically searched PubMed, EMBASE, Cochrane Central Register of Controlled Trials, and ClinicalTrials.gov databases up to April 4, 2023, using terms such as: ‘Type 1 Diabetes’, ‘T1DM’, ‘closed-loop’, ‘automated insulin delivery’, ‘AID’, ‘randomized’ and ‘RCT’. The complete search strategy is available in Supplementary Appendix A. No filters or language restrictions were applied in our search. Grey literature was not searched. We also utilised a technique of backward snowballing, searching for additional eligible studies through a review of the references from prior publications [17]. Three authors performed the literature search independently (AG, AM, and LH) following predefined search criteria. Eventual conflicts were resolved by consensus among the authors.

### Study selection

The research question was defined according to the PICOTT framework and studies were included in the systematic review if they met the following eligibility criteria: (1) enrolling adult or paediatric patient population with T1DM; (2) comparing CL systems with UC; (3) assessing any of the outcomes of interest; (4) RCTs with parallel or crossover designs; and (5) with a minimum duration of at least 12 weeks. We included both hybrid-loop and fully CL systems in our analysis. UC was considered to include SAP, MDII, CGM, or PLGS. A full description of the current insulin devices can be found in Additional file 1: Table S1.

We excluded studies with overlapping patient populations, understood as derived from overlapping institutions, patients and recruitment periods, and clinical trials with no results after contacting the primary investigator. Additionally, crossover studies with less than 12 weeks of washout periods were excluded from the analysis of change in HbA1c (%), unless outcomes from each phase of the study were reported. In this case, only phase 1 results were included in our HbA1c analysis. If two or more studies with overlapping populations reported different outcomes of interest, they were included if these could be analysed in a non-overlapping manner.

### Data collection and extraction

Two authors (AG and EMHP) extracted outcome data independently using a standardised document and disagreements were resolved by consensus. Four corresponding authors were contacted for additional data (one provided the information). Furthermore, three independent authors (IRM, VCSM and ACS) extracted additional baseline data for individual studies, including study and patient characteristics (Tables 1, 2). Participant-level data was not requested.

**Table 1:** Baseline qualitative characteristics of included studies

Study	NCT ID	Country	Intervention	Control	Population	Primary outcome	Outcomes measured^a^
Abraham [26]	ACTRN12616000753459	Australia	MiniMed 670G	MDI/SAP therapy	Adults and Children	TIR 70–180 mg/dL	Clinical and functional
ADAPT (Choudhary [27])	NCT04235504	France, Germany And UK	MiniMed 670G	MDI therapy	Adults	Change in HbA1c	Clinical and functional
APCam11 (Tauschmann [28])	NCT02523131	USA and UK	Modified FlorenceM	SAP therapy	Adults and children	TIR 70–180 mg/dL	Clinical and functional
AP@home (Thabit [29])	NCT01778348 + NCT01961622	UK, Germany and Austria	FlorenceD2A and FlorenceD2W	SAP therapy	Adults and children	TIR 70–180 mg/dL for adults and TIR 70–145 mg/dL for children and adolescents	Clinical
Boughton [30]	NCT04025762	UK and Austria	CamAPS FX	SAP therapy	Adults	TIR 70–180 mg/dL	Clinical
Brown [31]	NCT03591354	USA	t:slim X2 with control-IQ	PLGS therapy	Adults	TIR 70–180 mg/dL	Clinical
Burnside [32]	ACTRN12620000034932	New Zealand	AndroidAPS 2.8	SAP	Adults and children	TIR 70–180 mg/dL	Clinical
DAN05 (Ware [33],Hood [46])	NCT02925299	UK and USA	FlorenceM or CamAPS FX	SAP therapy	Children	Change in HbA1c	Clinical and functional
DAN06 **(**Boughton [34])	NCT02871089	UK	FlorenceM/CamAPS FX	MDI therapy	Children and adolescents	AUC for the plasma C-peptide	Clinical
DCPL3 (Brown [35], Kudva [47])	NCT03563313	USA	t:slim X2 with Control-IQ	SAP therapy	Adults and children	TIR 70–180 mg/dL	Clinical and functional
DCLP4 (Pinsker [36])	NCT04436796	USA	Interoperable artificial pancreas system (iAPS)	SAP/PLGS therapy	Adults	TIR 70–180 mg/dL	Clinical and functional
DCLP5 (Breton [37], Cobry [48])	NCT03844789	USA	t:slim X2 with control-IQ	SAP/PLGS therapy	Children	TIR 70–180 mg/dL	Clinical and functional
DIABELOOP WP7 (Benhamou [38])	NCT02987556	France	Diabeloop Generation 1 (DBLG1)	SAP therapy	Adults	TIR 70–180 mg/dL	Clinical and functional
iDCL (Kovatchev [39])	NCT02985866	USA	Control-IQ	SAP therapy	Adults	Time below 70 mg/dL and above 180 mg/dL	Clinical
Garg [40]	NCT02748018	USA and Canada	MiniMed 670G hybrid closed loop	CSII	Children, adolescents and adults	Group 1: change in HbA1c Group 2: reducing %TBR < 70 mg/dL	Clinical and functional
Matejko [7]	NCT04616391	Poland	MiniMed 780G	MDI therapy	Adults	TIR 70–180 mg/dL	Clinical and functional
McAuley [41]	ACTRN12617000520336	Australia	MiniMed 670G	MDI/SAP therapy	Adults	TIR 70–180 mg/dL	Clinical and functional
ORACL (McAuley [42])	ACTRN126190000516190	Australia	MiniMed 670G	SAP therapy	Adults	TIR 70–180 mg/dL	Clinical and functional
PEDAP trial (Wadwa) [43])	NCT04796779	USA	T:slim X2 with Control-IQ	MDI/SAP therapy	Children	TIR 70–180 mg/dL	Clinical
Reiss [44]	NCT03428932	USA	MiniMed 670G	MDI/SAP therapy	Children	Metrics of gray matter	Clinical and functional
Russell [11]	NCT04200313	USA	iLet device	PLGS/SAP/MDI therapy	Adults and children	Change in HbA1c	Clinical
Ware [45]	NCT03784027	Austria, Germany, Luxembourg and UK	CamAPS FX	SAP therapy	Children	TIR 70–180 mg/dL	Clinical

*CGM *Continuous glucose monitor*, TIR *Time in Range*, MDI *Multiple Daily Injections*, SAP *sensor augmented pump*, AUC *Area under the curve*, PLGS, *Predictive low-glucose suspend system*, HbA1c *Glycated Hemoglobin*, UK *United Kingdom*, USA *United States of America

^*a*^Functional outcomes include participant-reported questionnaires/patients reported outcomes

**Table 2:** Baseline quantitative characteristics of included studies

Study	Patient No, I/C	Female %, I/C	Age, years, I/C^d^	Duration of assessment, wks	Study design	Baseline HbA1c, % (mmol/mol)^d^	Duration of diabetes, years^d^	BMI, kg/m^2d^	Baseline daily insulin dose, units/kg^d^
Abraham [26]	67/68	55/57	15.2 ± 3.3/15.4 ± 3.0	24	Parallel	8.0 ± 1.0 (64 ± 10)/7.9 ± 1.0 (63 ± 11)	7.9 ± 4.2/7.6 ± 3.4	0.7 ± 0.8/0.7 ± 0.7^c^	0.8 ± 0.2/0.9 ± 1.2
ADAPT (Choudhary [27])	41/41	54/39	41.5 ± 11.63/39.7 ± 13.12	24	Parallel	9.0 ± 1.0 (75.7 ± 7.83)/9.1 ± 0.7 (74.9 ± 10.64)	18.8 ± 11.4/18.1 ± 10.0	27.0 ± 4.4/25.8 ± 4.9	54.3 ± 25.9/53.3 ± 22.3^e^
APCam11 (Tauschmann [28])	46/40	48/55	22 (13–36)/21 (11–36)^a^	12	Parallel	8.3 ± 0.6(68 ± 7)/8.2 ± 0.5 (66 ± 5)	13 (7–20)/10 (7–19)^a^	28 ± 4)/27 ± 3	0.76 ± 0.25/0.69 ± 0.8
AP@home (Thabit [29])^b^	33/ 25	45/44	40.0 ± 9.4/12.0 ± 3.4	12	Crossover	8.5 ± 0.7 (69 ± 7)/8.1 ± 0.9 (65 ± 10)	20.9 ± 9.3/4.7 ± 2.6	25.5 ± 4.4/18.9 ± 3.5	0.62 ± 0.15/0.89 ± 0.24
Boughton [30]	20/17	40/47	68(63–70)/67 (62–70)^a^	16	Crossover	7.5 ± 1.0 (57 ± 10)/7.4 ± 0.9 (58 ± 10)	38 (32–48)/38 (32–48)^a^	28.2 (25.4–31.7)/27.4 (24.9–38.5)^a^	45·8 (38.3–51.1)/40·0 (35·4–62·4)^e^
Brown [31]	54/55	52/45	32 ± 14/34 ± 17	13	Parallel	7.0 ± 0.8 (54 ± 8.5)/7.1 ± 0.8 (54 ± 8.4)	18 ± 8.3/16 ± 7.3	26 (23, 30)/25 (23, 29)^a^	0.59 (0.49, 0.86)/0·68 (0.46, 0.93)^a^
Burnside [32]	44/53	52/48	26.59 ± 14.33/23.29 ± 17.51	24	Parallel	7.55 (60.0 ± 13.7)/7.65 (62.1 ± 9.1)^f^	15.20 ± 13.42/12.33 ± 11.82	24.40 ± 5.86/23.67 ± 6.42	44.6 ± 16.17/43.01 ± 17.36
DAN05 (Ware [33], Hood [46]	65/68	57/57	13.1 ± 2.6/12.8 ± 2.9	24	Parallel	8.2 ± 0.7 (66 ± 8)/8.3 ± 0.8 (67 ± 8)^c^	6.3 ± 3.3/6.6 ± 3.1	0.35 ± 0.86/0.58 ± 0.89^c^	0.93 ± 0.23/0.95 ± 0.24
DAN06** (**Boughton [34])	51/46	49/39	12 ± 2/12 ± 2	96	Parallel	10.7 ± 1.8 (93 ± 18)/10.5 ± 1.6 (94 ± 20)	NA	53 ± 29/51 ± 34^g^	0.87 ± 0.33/0.82 ± 0.38
DCPL3 (Brown [35], Kudva [47])	112/56	48/54	33 ± 16/33 ± 17	24	Parallel	7.4 ± 1.0/7.4 ± 0.8	17 (8, 28)/15 (7, 23)^a^	25 (23, 29)/25 (22, 28)^a^	46 (31, 62)/45 (35, 61)^a, e^
DCLP4 (Pinsker 2022) [36])	18/16	36·8/56·3	41 ± 16/37 ± 15	13	Crossover	6.9 ± 1.0	18 (12, 29)^a^	28 ± 5	NA
DCLP5 (Breton [37], Cobry [48])	78/23	49/52	11.3 ± 2.0/10.8 ± 2.4	16	Parallel	7.7 ± 1.1/8.0 ± 1.1	5.0 ± 2.8/6.0 ± 2.8	0.4 ± 1·0/0.5 ± 1·0^c^	0.89 ± 0.24/0.94 ± 0.24
DIABELOOP WP7 (Benhamou [38])	32/31	62	48.2 ± 13.4	12	Crossover	7.6 ± 0.9 (59.4 ± 9.8)	28.0 ± 13.6	24.8 ± 3.5	36.3 ± 8.9^e^
iDCL (Kovatchev [39])	65/62	49/45	33 ± 16/32 ± 14	12	Parallel	7.4 ± 0.9 (57 ± 9.8)/7.4 ± 0.8 (57 ± 8.7)	19 (7, 27)/16 (11, 27)^a^	27 (24, 31)/25 (23, 29)^a^	0.73 ± 0.22/0.68 ± 0.25
Garg [40]	151/151	47/62	39.9 ± 19.8/35.7 ± 18.4	24	Parallel	8.2 ± 1.3/8.1 ± 1.2	21.5 ± 13.6/19.6 ± 13.1	26.8 ± 5.8/27.0 ± 6.9	NA
Matejko [7]	20/17	40/47·1	39.8 ± 8.3/40.9 ± 7.8	12	Parallel	7.05 ± 0.8 (54 ± 9)/7.4 ± 1.2 (57 ± 13)	17.1 ± 12.2/17.6 ± 12.2	24.5 ± 3.3/25.6 ± 2.64	NA
McAuley 2020 [41]	61/59	54/53	43.7 ± 11.7/44.7 ± 11.8	26	Parallel	7.4 ± 0.9 (62 ± 12)/7.5 ± 0·8 (61 ± 10)	24.0 ± 12.0/24.1 ± 12.5	26.8 ± 5.3/26.0 ± 4.0	0.51 (0.41, 0.63)/0.54 (0.45, 0.66)^a^
ORACL (McAuley [42])	15/15	63	67 ± 5	16	Crossover	7.6 ± 0.9 (58 ± 7)	38 (20–47)^a^	27.6 (26.4–31.0)^a^	0.55 (0.41–0.66)^a^
PEDAP trial (Wadwa [43])	68/34	49/56	3.84 ± 1.23/4.06 ± 1.25	13	Parallel	7.5 ± 1.2/7.7 ± 0.9	1.04 (0.71·1.85)/1.40 (0.91,2·11)	81 (57,94)/77 (56,9)^g^	0.66 ± 0.17/0.66 ± 0.23
Reiss [44]	21/21	40/47	14–17	24	Parallel	8.7/ 8.45^f^	NA	NA	NA
Russell [11]	147/72	49/38	28 ± 19/28 ± 20	13	Parallel	7.9 ± 1.2/7.7 ± 1·1	16 ± 14/18 ± 15	28.9 ± 5.5/29.1 ± 6.9	0.75 (0.57, 1.00)/0.75 (0.56, 0.94)^a^
Ware [45]	39/35	54/29	5.6 ± 1.4/5.6 ± 1.7	16	Crossover	7.3 ± 0.7 (56.3 ± 7.4)/7.4 ± 0.6 (57 ± 7.1)	2.5 ± 1.7/2.7 ± 1.9	67.3 ± 23.2/71.1 ± 24.6^g^	0.76 (0.67–0.83)/0.77 (0.69–0.86)^a^

*NA *not available*, I/C *Intervention/Control*, HbA1c, *Glycated Hemoglobin

^a^Median (IQR)

^b^Data are reported as *Adults/Children and Adolescents*

^c^Reported as Body-mass index Z score

^d^Reported values as mean ± SD unless specified

^e^Values are reported in units per day, not units/kg

^f^No SD available

^g^Age and sex-adjusted BMI percentile

For studies reporting data for paediatric and adult patients separately, we planned to analyse these as separate comparisons. For crossover studies, we planned a priori to analyse group means and standard deviations, assuming no correlation between groups (as parallel study designs). The bias introduced with this assumption is generally conservative [18]. For missing means data, we used the formula proposed by Wan et al. [19] using medians and interquartile ranges as recommended by the Cochrane Collaboration [18]. We collected adjusted mean differences (MD) as originally reported in each study when available.

### Outcome measurements

Our main outcomes were TIR 70-180 mg/dL as the primary outcome and HbA1c (%) change. Secondary outcomes of interest included coefficient of glucose variability (CV), % time < 70 mg/dL, % time < 54 mg/dL, nocturnal hypoglycaemia (< 70 mg/dL), and %time > 250 mg/dL. We assessed the following psychosocial outcomes: Hypoglycaemia Fear Survey (HFS) [20]; Diabetes Treatment Satisfaction Questionnaire (DTSQ) [21]; treatment distress measured by the scales Diabetes Distress Scale (DDS) [22] and Problem Areas in Diabetes (PAID) [23]. Safety endpoints included diabetic ketoacidosis (DKA) and severe hypoglycaemia.

### Quality assessment

Each included study was appraised using the Cochrane Risk of Bias Assessment Tool (RoB-2) for RCTs [24] by at least two independent investigators (AG, CH, IS, and CG). Further, the Grading of Recommendations, Assessment, Development and Evaluation (GRADE) tool was employed by two independent authors (IAM and IRM) using the GRADEpro Guideline Development Tool [25] to evaluate the level of certainty of the evidence in this meta-analysis, with categorizations ranging from high to very low [26]. Any disagreements were discussed and resolved through a consensus.

### Statistical analysis

Binary adverse outcomes were summarised using the Mantel–Haenszel test, with an odds ratio (OR) and 95% confidence interval (CI) as a measure of effect size. Continuous outcomes were compared with weighted and standardised MDs. Statistical heterogeneity was assessed by I^2^ and sources of heterogeneity were sought if I^2^ was greater than 50%. When low heterogeneity was identified (I^2^ < 25%), a fixed-effects model was used. We performed sensitivity analyses using the leave-one-out strategy as well as Baujat plots. We further investigated causes of heterogeneity by performing subgroup analyses according to type of AID device.

In addition, a random effect meta-regression analysis was performed to assess the impact of baseline HbA1c and study duration on overall MD. Publication bias was assessed for HbA1c and TIR 70–180 mg/dL through the generation of a funnel plot and Egger’s test, where a p-value less than 0.05 indicates the presence of publication bias. Review Manager 5.4.1 software (Nordic Cochrane Centre, The Cochrane Collaboration, Copenhagen, Denmark) and RStudio version 4.1.2 (R Foundation for Statistical Computing) were used for the statistical analysis.

### Role of the funding source

There was no funding source for this study. AG and EMHP had full access to all the data in the study and all authors had responsibility for the final publication.

## Results

**Fig. 1:**
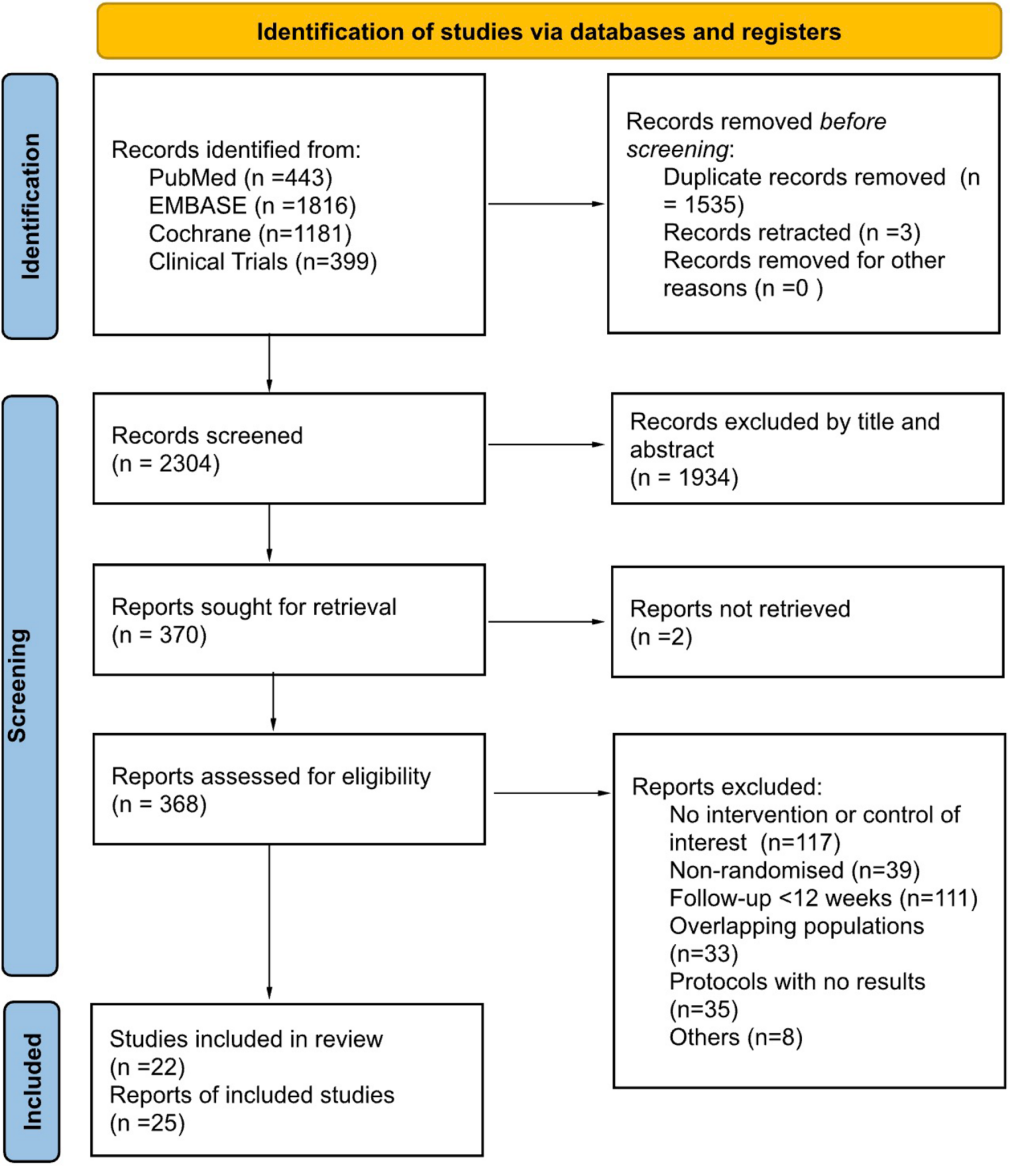
PRISMA flow of study selection

Our search identified a total of 3839 unique studies, of which 25 reports from 22 RCTs, including 2376 randomised participants, fulfilled the study eligibility criteria (Fig. 1) [27]. Of the 25 reports identified, 22 assessed primarily clinical outcomes [8, 12, 28–47], while 3 studies [48–50] assessed solely patient-reported outcomes.

### Characteristics of included studies

Characteristics of studies contributing data to this meta-analysis are presented in Tables 1 and 2. The trials were conducted across eight countries spanning three continents. Seventeen studies had a parallel-group design, while five were crossover studies. Most RCTs included only adults (n = 9), while a similar number included only children (n = 6) or mixed both adults and children (n = 7). Females comprised 46.6% (n = 1068) of the included population. The mean age of adult participants ranged from 32 to 68 years, and of paediatric participants ranged from 3.8 to 15.4 years. The mean duration of T1DM ranged from 1 to 38 years, with a mean Body Mass Index ranging from 18.9 to 29.1 kg/m^2^, and a baseline HbA1c ranging from 6.9 to 10.7%. Among the 19 included trials, four (n = 380) assessed the use of CamAPS FX (CamDiab) [31, 36, 38, 46]; five (n = 636) assessed MiniMed 670G (Medtronic) [28, 34, 43, 44, 46]; five (n = 605) assessed t:slim X2 with Control IQ (Tandem) [33, 35, 36, 38, 45]; two (n = 144) assessed Modified Florence [30, 31]; two (n = 119) assessed MiniMed 780G (Medtronic) [8, 29]; two assessed openAPS (n = 129) [34, 41]; one (n = 63) assessed DBLG1 (Dbl-diabetes) [40]; and one (n = 219) assessed iLet Bionic Pancreas (Beta Bionics) [12]. Duration of CL or UC use ranged from 12 to 96 weeks.

### Effects on glucose control

**Fig. 2:**
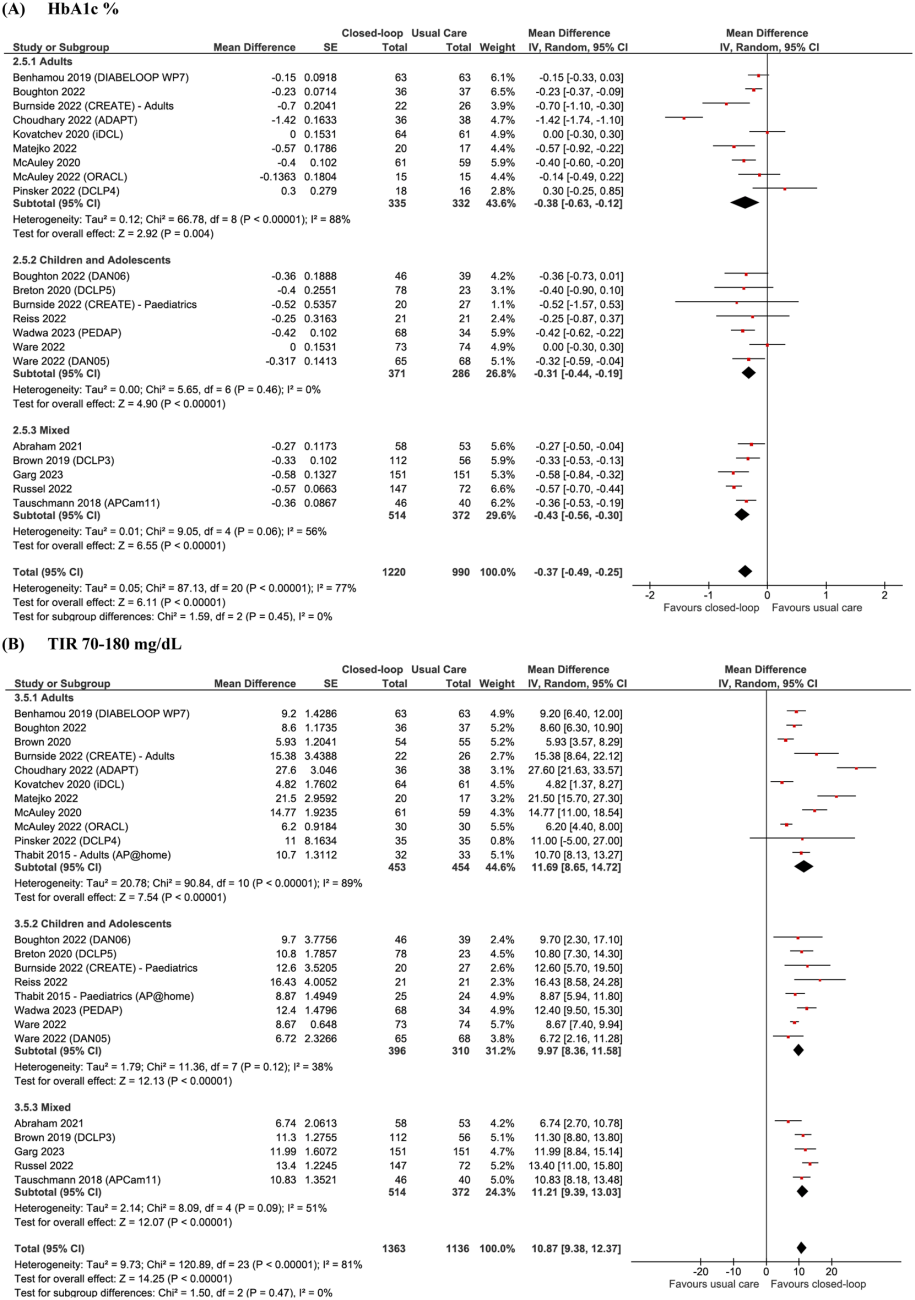
Forest plots for (**A**) HbA1c % and (**B**) TIR 70–180 mg/dL

In a pooled analysis of 19 studies (n = 2210) for the primary outcome displayed in Fig. 2A and Table 3, treatment with CL systems led to a significant decrease in HbA1c % (MD – 0.37; 95% CI − 0.49 to − 0.26; p < 0.0001) and mmol/mol (MD − 4.77; 95% CI − 6.39 to − 3.14;p < 0.001), for adults (MD − 0.38; 95% CI − 0.63 to − 0.12; p = 0.004), children (MD − 0.31; 95% CI − 0.44 to − 0.19; p < 0.001) and mixed populations (MD − 0.46; 95% CI − 0.56 to − 0.30; p < 0.0001). There was a high statistical heterogeneity for the overall (I^2^ = 77%), adult (I^2^ = 88%), and mixed analyses (I^2^ = 62%), but not for children (I^2^ = 0%). In the overall analysis of 22 studies (n = 2499), there was also a significant 10.87% increase in TIR 70–180 mg/dL for the CL group when compared to UC (95% CI 9.38 to 12.37; p < 0.0001; Fig. 2B), which was similarly seen in adults (MD 11.69; 95% CI 8.65 to 14.62; p < 0.0001), children (MD.9·97; 95% CI 8.36 to 11.58; p < 0.0001) and mixed populations (MD 11.21; 95% CI.9·39 to 13.03; p < 0.0001). Statistical heterogeneity was high (I^2^ = 81%), but decreased in the children subgroup (I^2^ = 38%).

**Table 3:** Summary results of overall meta-analysis for each outcome and according to age subgroups

Outcome	No of patients (no of comparisons)	Pooled Result (CI 95%)	P value	Heterogeneity
HbA1c (%)^a^				
Overall	2210 (20)	− 0.37 (− 0.49 to − 0.26)	< 0.001	77%
Adults	667 (9)	− 0.38 (− 0.63 to − 0.12)	0.004	88%
Pediatric	657 (7)	− 0.31 (− 0.44 to − 0.19)	< 0.001	0%
Mixed	886 (4)	− 0.46 (− 0.56 to − 0.30)	< 0.001	62%
HbA1c (mmol/mol)^a^				
Overall	1229 (13)	− 4.77 (− 6.39 to − 3.14)	< 0.001	85%
Adults	596 (7)	− 4.87 (− 7.66 to − 2.97)	0.001	91%
Pediatric	327 (3)	− 6.77 (− 11·90 to − 1·64)	0.010	83%
Mixed	306 (6)	− 3.70 (− 5.00 to − 2.39)	< 0.001	0%
TIR^a^				
Overall	2499 (24)	10.87 (9.38 to 12.37)	< 0.001	81%
Adults	907 (11)	11.69 (8.65 to 14.72)	< 0.001	89%
Pediatric	706 (8)	9.97 (8.36 to 11.58)	< 0.001	38%
Mixed	886 (5)	11.21 (9.39 to 13.03)	< 0.001	51%
DKA^b^				
Overall	2413 (22)	1.62 (0.64 to 4.12)	0.31	0%
Adults	1020 (11)	0.85 (0.22 to 3·25)	0.81	0%
Pediatric	669 (7)	3.06 (0.63 to 14.85)	0.17	0%
Mixed	724 (4)	2.67 (0.11 to 67.40)	0.55	NA
Severe hypoglycaemia^b^				
Overall	2244 (22)	1.29 (0.78 to 2.15)	0.32	0%
Adults	915 (11)	1.24 (0.64 to 2.38)	0.53	0%
Pediatric	773 (8)	2.35 (0.89 to 6.20)	0.08	0%
Mixed	556 (3)	0.11 (0.01 to 2.03)	Not estimable	NA
Pro				
Distress^c^	763 (7)	− 0.18 (− 0.34 to − 0.03)	0.02	8%
FOH^b^	403 (5)	− 2·35(− 5·21 to 0·51)	0.11	45%
Satisfactionª	569 (6)	0.00 (− 3.10 to 3.10)	0.83	79%
% Time (< 54 mg/dl)^a^				
Overall	1917 (19)	− 0.14 (− 0.22 to − 0.07)	< 0.001	77%
Adults	842 (10)	− 0.23 (− 0·37 to − 0·10)	< 0.001	85%
Pediatric	577 (6)	− 0·03 (− 0·09 to − 0·03)	0.32	0%
Mixed	498(3)	− 0.15 (− 0.31 to 0.02)	0.08	83%
% Time (< 70 mg/dl)^a^				
Overall	2499 (24)	− 0.65 (− 1.05 to − 0.26)	0.001	95%
Adults	907 (11)	− 0.82 (− 1·43 to − 0·21)	0.008	96%
Pediatric	706 (8)	− 0.14 (− 0.41 to 0.68)	0.62	83%
Mixed	886 (5)	− 1.39 (− 2·17 to − 0·60)	< 0.001	92%
% Time (> 250 mg/dl)^a^				
Overall	1731 (16)	− 4.46 (− 5.79 to − 3.14)	< 0.001	93%
Adults	769 (9)	− 3.51 (− 4.97 to − 2.05)	< 0.001	90%
Pediatric	464 (4)	− 6.63 (− 8.14 to − 4.92)	0.009	32%
Mixed	498 (3)	− 4.24 (− 9.16 to 0.67)	0.09	97%
Nocturnal hypoglycaemia^a^				
Overall	1661 (17)	− 1.28 (− 1.76 to − 0.79)	< 0.001	84%
Adults	717 (9)	− 1.03 (− 1.70 to − 0.36)	0.003	87%
Pediatric	474 (6)	− 1.18 (− 1.91 to − 0·45)	0.002	55%
Mixed	470 (2)	− 3.17 (− 7.37 to 1.03)	0.14	96%
CV^a^				
Overall	2197 (23)	− 1.09 (− 1.80 to − 0.39)	< 0.001	81%
Adults	907 (11)	− 1.74 (− 2.79 to − 0.70)	0.002	83%
Pediatric	706 (8)	0.33 (− 0.88 to 1.55)	0.88	77%
Mixed	584 (4)	− 1.81 (− 3.38 to − 0·25)	0.02	82%

*TIR* time in range, *PRO* Patients-Reported Outcomes, *FOH* Fear of hypoglycaemia, *HP* hyperglycemia, *CV* coefficient of variation

^a^Mean difference, ^b^Odds ratio, ^c^Standardized mean difference

**Fig. 3:**
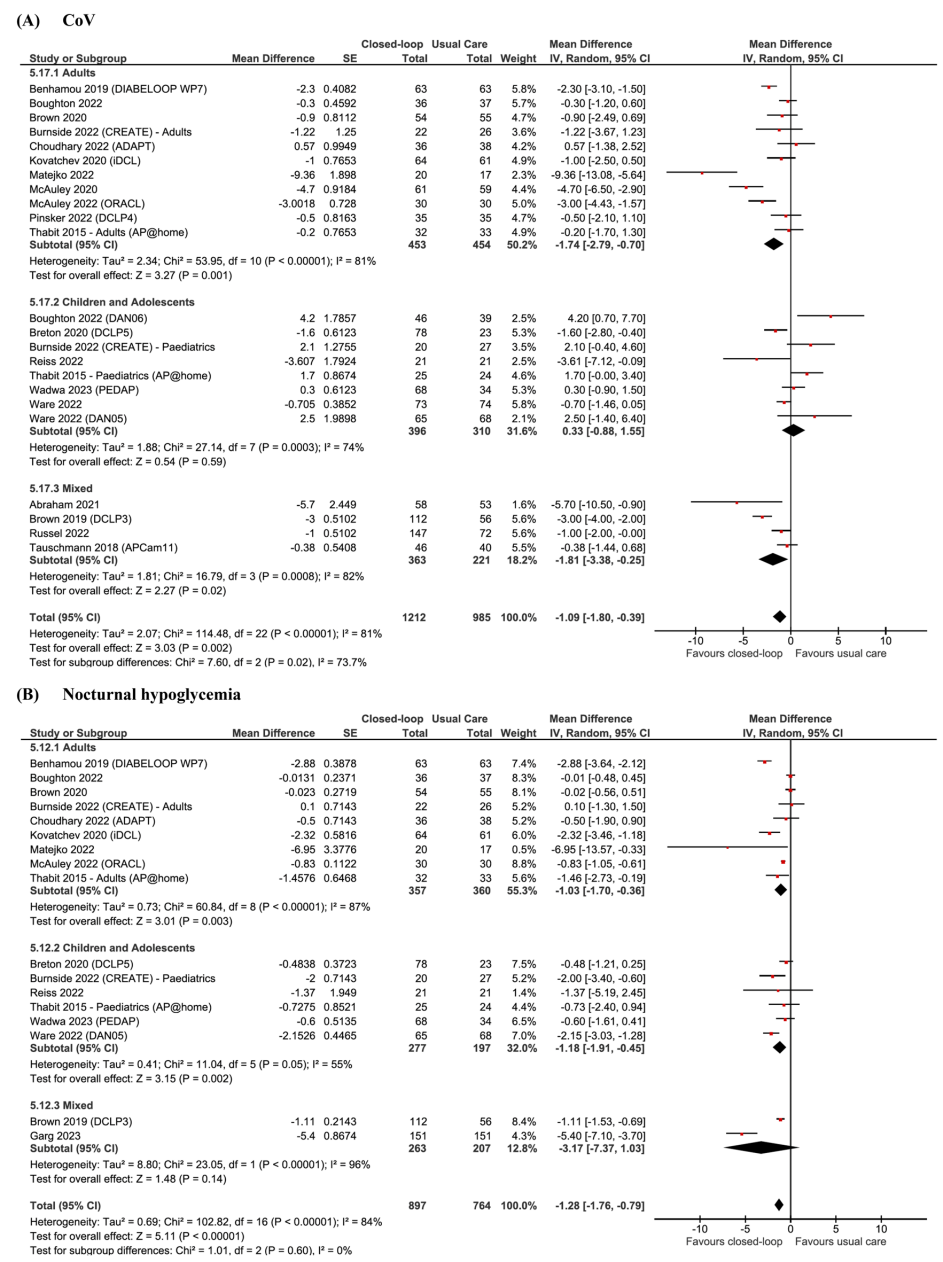
Forest plots for (**A**) CV and (**B**) nocturnal hypoglycaemia

**Fig. 4:**
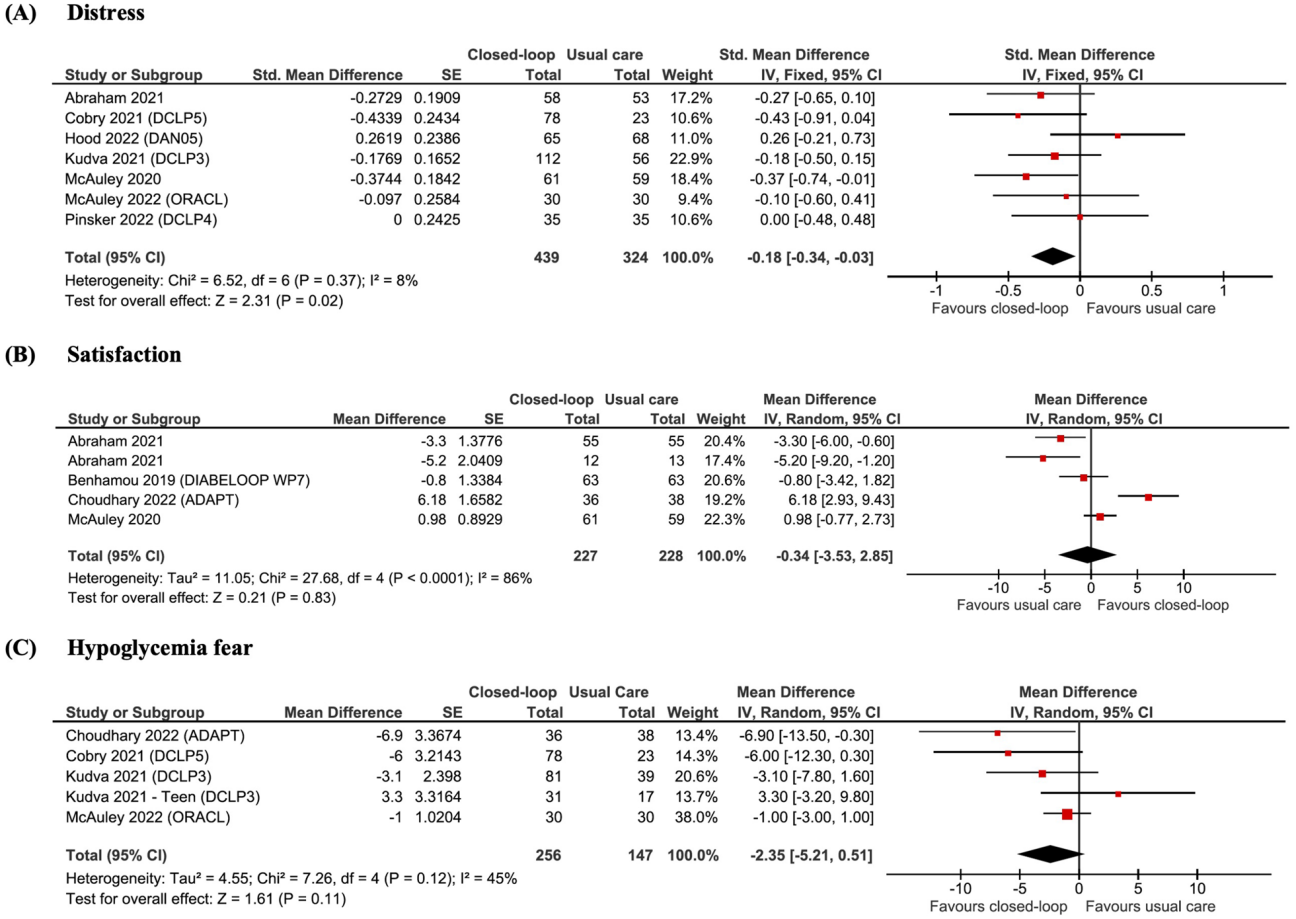
Meta-analysis of patient-reported outcomes of (**A**) diabetes distress measured by Diabetes Distress Survey (DDS) and Problem Areas in Diabetes (PAID), **B** Diabetes Treatment Satisfaction Questionnaire (DTSQ), and (**C**) Hypoglycaemia Fear Scale (HFS)

Further analyses for glycaemic control significantly favoured the use of CL systems for endpoints of CV (MD − 1.09; 95% CI − 1.80 to − 0.39; p = 0.0007; Fig. 3A), % time < 70 mg/dL (MD − 0.65; 95% CI − 1.05 to − 0.26; p = 0.009), % time < 54 mg/dL (MD − 0.14; 95% CI − 0.22 to − 0.07; p < 0.0001), % time > 250 mg/dL (MD − 4.46; 95% CI − 5.79 to − 3.14; p < 0.0001), and nocturnal hypoglycaemia (MD − 1.28; 95% CI − 1.76 to − 0.79; p < 0.0001; Fig. 3B). No significant differences were found for the use of CL in children for % time < 54 mg/dL (p = 0.32); < 70 mg/dL (p = 0.62); and CV (p = 0.88) when compared to UC. Further detailed findings for age subgroups can be seen in Table 3. As shown in Fig. 4, the rate of episodes of DKA (Additional file 1: Figure S1A) and severe hypoglycaemia (Additional file 1: Figure S1B) was not significantly different between groups (p = 0.31 and p = 0.32, respectively).

### Effects on psychosocial outcomes

The pooled analysis for patient-reported outcomes found decreased diabetes distress for the CL group (SMD − 0.18; 95% CI − 0.34 to − 0.03; p = 0.02; Fig. 4A), but no significant differences for fear of hypoglycaemia (p = 0.11, Fig. 4B) and treatment satisfaction (p = 0.83, Fig. 4C).

### Risk of bias in included studies

The risk of bias assessment of each RCT is provided in the Additional file 1: Appendix A for clinical (Additional file 1: Figure S3) and functional (Additional file 1: Figure S4) outcomes. For clinical outcomes, three were rated as “some concerns'' due to missing outcome data [7] and deviations from the protocol (machine errors) [35, 40], and seven were rated as “high risk” due to lack of laboratory-measured HbA1c assessment [44, 46] or due to insufficient washout time [36, 38, 43, 47] in crossover studies. All trials were open-label but used adequate methods for allocating participants and objective measurements of clinical outcomes. For patient-reported outcomes, trials were assessed as “some concerns'' due to the subjective nature of the assessment (Additional file 1: Figure S4).

### GRADE assessment and publication bias

Following the GRADE criteria (Additional file 1: Table S3), there was moderate certainty of evidence for HbA1c reduction in the mixed and paediatric populations, and for TIR 70–180 mg/dL in the paediatric population. In contrast, there was low certainty of evidence for HbA1c reduction in the adult population, for TIR 70–180 mg/dL in the mixed and adult populations, and for CV and night hypoglycaemia. Funnel plots for HbA1c showed no indication of publication bias visually (Additional file 1: Figure S5) or based on Egger’s regression test (p = 0.93; Additional file 1: Figure S6A), yet a significant value was found for TIR (p = 0.02; Additional file 1: Figure S6B).

### Sensitivity analyses

**Fig. 5:**
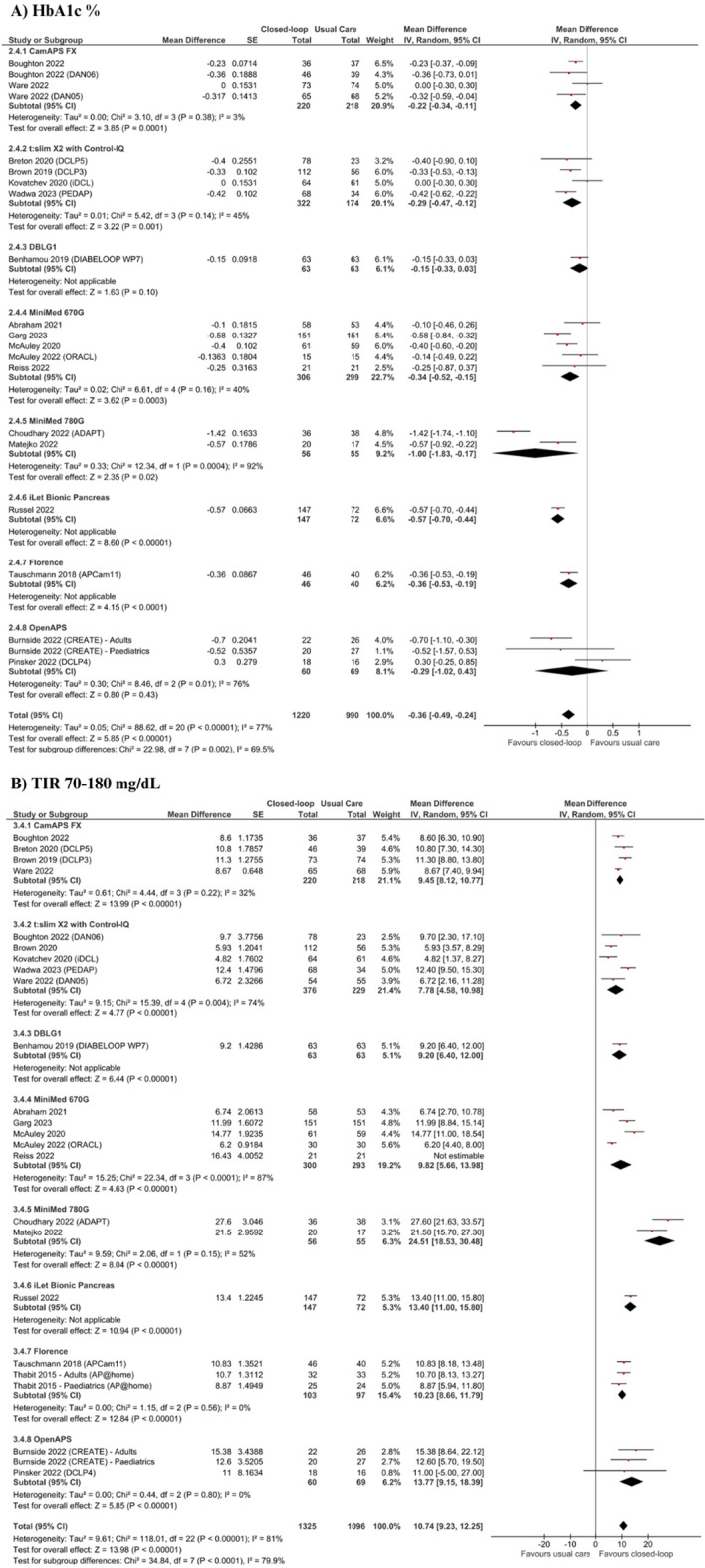
Subgroup analysis based on CL systems for (**A**) HbA1c % and (**B**) TIR 70–180 mg/dL

We explored the consistency of treatment effects using the leave-one-out strategy (Additional file 1: Figure S7), which revealed that Choudhary 2022 [29] was the study responsible for driving the heterogeneity from 58 to 77%, also confirmed by the Baujat plot (Additional file 1: Figure S8). Yet, results remained statistically significant to favour CL systems even when each individual study was removed from the analysis (Additional file 1: Figure S7). To further investigate reasons for the observed heterogeneity of effect for glycaemic control endpoints, we stratified our analyses by type of AID machines (Additional file 1: Table S2). As seen in Fig. 5, heterogeneity decreased substantially for most machine subgroups and findings remained mostly consistent with the overall analysis, favouring CL systems over UC. Nonetheless, the openAPS subgroup revealed no significant differences between CL and UC for change in HbA1c. MiniMed 780G and iLet Pancreas were found to be most effective to improve HbA1c and TIR outcomes (Fig. 5), MiniMed 670G was most effective to improve CV (Additional file 1: Figure S1), and openAPS was most effective at preventing nocturnal hypoglycaemia when compared to other machines (Additional file 1: Table S2). In addition, we performed a meta-regression based on follow-up duration and baseline HbA1c (Additional file 1: Figure S5). Although the results showed no significant association between the study duration and the mean differences for change in HbA1c (p = 0.57; Additional file 1: Figure S9), higher baseline HbA1c was significantly associated with greater change scores (p = 0.02; Additional file 1: Figure S10).

## Discussion

In this systematic review and meta-analysis of 22 RCTs and 2376 patients, we compared the use of AID devices versus UC during a period of 12 to 96 weeks. Our main findings were: (1) A significantly improved HbA1c level, % TIR 70–180 mg/dL, CV, % time < 54 mg/dL, < 70 mg/dL, < 250 mg/dL and risk of nocturnal hypoglycaemia, with the use of AID devices; (2) a significant improvement in diabetes distress in the CL group; (3) no significant difference in the risk of DKA or severe hypoglycaemia between groups; (4) no significant reduction in % time < 54 mg/dL, < 70 mg/dL, and CV observed between paediatric groups, and (5) no significant improvement in fear of hypoglycaemia and treatment satisfaction.

Achieving glycaemic control of T1DM while also avoiding hypoglycaemia is a challenge for patients [51, 52]. A high cognitive load for T1DM patients and care team is required and previous studies show distress or depressive symptoms in up to 40% of patients [53]. Although HbA1c is currently the metric of choice by most endocrinology and diabetes societies [54, 55], TIR and HbA1c should be used as complementary parameters to guide care [56] and allow evaluation in clinical research [57].

To our knowledge, our study is the most comprehensive meta-analysis of use of AID for 12–96 weeks. Our analysis integrated data from 25 reports and 2376 participants, a population that almost tripled compared to a previous meta-analysis [14]. Furthermore, this is the first analysis with studies over 12 weeks of duration, stratified by age groups and type of AID device used. Our findings augment the certainty about the beneficial effects of the continuous use of CL systems on HbA1c, TIR, hypoglycaemia, and distress of patients, without increasing the risk of AEs. Given that glycaemic variability has been linked to chronic diabetic complications [58], respective reductions of 0·37% (4.77 mmol/mol) in HbA1c levels and 1·09% in CV have important implications for patient care. As the mean baseline HbA1c in our population was 7·73% (61 mmol/mol), our findings present a conservative and safe strategy to avoid the risk of hypoglycaemia commonly associated with large changes in HbA1c [59]. Furthermore, an increase of 10% TIR has been correlated with an HbA1c reduction of 0·5–0·8% [60], which is slightly higher compared to our TIR and HbA1c assessment. Our analyses also show that higher HbA1c levels at baseline are correlated with greater changes in HbA1c after the use of such devices, which may lead to further benefits to certain patient groups. Our findings are similar to the analyses by Weinsman and colleagues [13], although our results for reduction of time in hypoglycaemia are much smaller. The longer periodicity of the studies included provides a pragmatic setting for assessment, where greater variables and confounding factors reflect a better real-life picture of treatment impact.

In addition, our meta-analysis provides a unique framework for comparing 7 permutations of different technologies. The breadth of these findings provides estimates of treatment effects with particular relevance to clinical decision-making and cost-effectiveness analyses. The application of our results may be illustrated through an approach to device selection. For example, some devices appeared to offer the greatest potential for improved glycaemia compared to other systems in our sensitivity analyses, although no definite conclusions can be as no head-to-head comparisons were performed. Furthermore, there is a growing body of literature assessing the use of openAPS, or “do-it-yourself” (DIY) devices which are remotely controlled by open-source algorithms [61]. Given the limited knowledge about DIY systems [62], our analysis provides insight into the potential benefit of openAPS.

Most studies in our analysis did not assess fully automated systems [8, 29, 31–38, 40–46], which still require manual input from the user [63]. Therefore, the use of such devices in children and adolescents remains a challenge. Previous meta-analyses on paediatric populations, such as a recent one by Michou and colleagues [64], have shown a reduced risk of hypoglycaemia when assessing RCTs of mostly less than 12 weeks duration. Nonetheless, our analysis with RCTs of 12 to 96 weeks duration did not show a significantly reduced risk of hypoglycaemia nor coefficient of variation for the paediatric population, which could have been due to several reasons. For instance, children are more likely to experience hypoglycaemia due to increased physical activity, hormonal changes, varied eating habits and lifestyle, and inability to communicate symptoms appropriately [65]. Furthermore, considerable proportion of RCTs included have reported system errors and malfunctioning during the longer duration of the trials, potentially having important impacts for children and adolescents who are at a higher risk of hypoglycaemia or those not achieving target control [4]. These findings have important implications to the design of future paediatric trials, which should consider placing significant focus on patient education, device functioning and type of system used.

Finally, this was the first meta-analysis to assess how long-term use of AID impacts patient-reported outcomes with a considerable number of studies. Although our findings show significantly improved diabetes distress and a tendency for reduced fear of hypoglycaemia, no benefits were seen for treatment satisfaction. The high cost of AID devices, connectivity problems, automation-related errors, pump glitches, and other issues associated with insulin pumps have been perceived as drawbacks by T1DM patients [5]. Moreover, most studies included in our analyses use CL algorithms that still require manual bolus input. Further improvements towards fully AID may result in improved quality of life and treatment satisfaction. Lastly, psychosocial measures varied between trials, limiting the populations of our analyses. Given that such outcomes have been recently receiving increased attention [5], future studies may consider using more consistent and widely used measures to aid interpretation of psychosocial impact.

Our study has important limitations. The lack of blinding in the studies, as it is potentially unfeasible to blind patients in such RCTs, reduced the certainty of evidence for our findings. It is important to note that heterogeneity was high for most glycaemic outcomes, especially in the adult and mixed populations. However, this finding was expected given the highly variable clinical and technical factors involved in studies performed in real-life conditions without supervision. Subgroup analyses of different machines and meta-regression were performed to minimise and interpret such heterogeneities. Furthermore, we did not search the grey literature, which can increase the risk of publication bias. However, we believe that restricting our research to peer-reviewed sources minimised other sources of bias ensuring a more rigorous evaluation. Unfortunately, no study used outcomes such as mortality or macrovascular and microvascular complications as outcomes. Therefore, our study relies on surrogate measures for patient-oriented outcomes. Finally, recent bihormonal CL systems were not included as the RCTs on these devices only had a short follow-up period.

## Conclusion

This systematic review and meta-analysis confirms previous findings in the literature of short-duration studies, showing that the prolonged use of AID devices under pragmatic settings results in a small, but important 0·37% (4.77 mmol/mol) reduction in HbA1c levels and may lead to a large 10·87% increase in TIR. Findings also suggest reductions in nocturnal and daily hypoglycaemia as well as patient distress without increasing the risk of DKA and severe hypoglycaemia. This estimate is beneficial in planning future long-term clinical trials assessing the use of fully automated and bihormonal AID devices. The synthesis of all system subgroups emphasises the potential benefits of certain CL systems, although this finding requires head-to-head comparisons before definitive conclusions can be made. Our results show that use of CL technology between 12 and 96 weeks has considerable benefits in a variety of clinical settings. Ultimately, it will be at the discretion of clinicians and patients to understand the potential benefits associated with different CL systems and decide on the most optimal insulin delivery method to improve patient outcomes.

### Supplementary Information

**Additional file 1: Appendix A.** Search Strategy. **Table S1.** Descriptions of current insulin delivery devices. **Table S2.** Additional glycemic outcomes based on CL machines. **Table S3. **GRADE assessment. **Figure S1.** Forest plots for (**A**) DKA and (**B**) severe hypoglycaemia. **Figure S2. **Subgroup analysis based on closed-loop system devices for the outcomes of (**A**) CV and (**B**) nocturnal hypoglycemia. **Figure S3.** Critical appraisal according to the Cochrane Collaboration’s tool for assessing risk of bias in randomised trials for clinical outcomes. **Figure S4. **Critical appraisal according to the Cochrane Collaboration’s tool for assessing risk of bias in randomised trials for functional outcomes. **Figure S5.** Funnel plots for (**A**) HbA1c % and (**B**) TIR 70-180 mg/dL show no evidence of publication bias. **Figure S6.** Egger’s regression test does not suggest significant publication bias for (**A**) HbA1c (%) endpoint; but suggests significant publication bias for (B) % TIR 70-180 mg/dL endpoint. **Figure S7.** Leave-one-out sensitivity analysis for the outcome of HbA1c (%). **Figure S8. **Baujat plot for the outcome of HbA1c (%). **Figure S9.** Meta-regression exploring the association between mean differences of HbA1c level (%) and duration of follow-up (weeks). **Figure S10.** Meta-regression exploring the association between mean differences of HbA1c level (%) and baseline HbA1c (%).

## Data Availability

All data are publicly available in the relevant primary and secondary papers from relevant trials as listed in the References.

## Abbreviations

AE: Adverse event
AID: Automated insulin delivery
CGM: Continuous glucose monitoring
CI: Confidence interval
CL: Closed-loop
CV: Coefficient of glucose variability
DDS: Diabetes distress scale
DIY: Do It Yourself
DKA: Diabetic ketoacidosis
DTSQ: Diabetes Treatment Satisfaction Questionnaire
HCL: Hybrid Closed-loop
HFS: Hypoglycaemia Fear Survey
MDII: Multiple daily insulin injections
OR: Odds ratio
PLGS: Predictive low-glucose suspend
PRISMA: Preferred Reporting Items for Systematic Reviews and Meta-analysis
PAID: Problem areas in diabetes
RCT: Randomised controlled trial
SAP: Sensor-augmented Insulin Pump
TIR: Time in range
T1DM: Type 1 diabetes mellitus
UC: Usual care
