# Supplementary material for: Glucose control and psychosocial outcomes with use of automated insulin delivery for 12 to 96 weeks in type 1 diabetes: a meta-analysis of randomised controlled trials
Source: Diabetol Metab Syndr. 2023 Sep 28;15:190. doi: 10.1186/s13098-023-01144-4 (PMC10537468; doi:10.1186/s13098-023-01144-4)
Supplement: Supplementary file 1 — Additional file 1: Appendix A. Search Strategy. Table S1. Descriptions of current insulin delivery devices. Table S2. Additional glycemic outcomes based on CL machines. Table S3. GRADE assessment. Figure S1. Forest plots for (A) DKA and (B) severe hypoglycaemia. Figure S2. Subgroup analysis based on closed-loop system devices for the outcomes of (A) CV and (B) nocturnal hypoglycemia. Figure S3. Critical appraisal according to the Cochrane Collaboration’s tool for assessing risk of bias in randomised trials for clinical outcomes. Figure S4. Critical appraisal according to the Cochrane Collaboration’s tool for assessing risk of bias in randomised trials for functional outcomes. Figure S5. Funnel plots for (A) HbA1c % and (B) TIR 70-180 mg/dL show no evidence of publication bias. Figure S6. Egger’s regression test does not suggest significant publication bias for (A) HbA1c (%) endpoint; but suggests significant publication bias for (B) % TIR 70-180 mg/dL endpoint. Figure S7. Leave-one-out sensitivity analysis for the outcome of HbA1c (%). Figure S8. Baujat plot for the outcome of HbA1c (%). Figure S9. Meta-regression exploring the association between mean differences of HbA1c level (%) and duration of follow-up (weeks). Figure S10. Meta-regression exploring the association between mean differences of HbA1c level (%) and baseline HbA1c (%). [file 13098_2023_1144_MOESM1_ESM.docx]

**SUPPLEMENTARY APPENDICES**

**For**

**Glucose Control and Psychosocial Outcomes with Prolonged Use of Automated Insulin Delivery in Type 1 Diabetes: A Meta-Analysis of Randomised Controlled Trials**

**Godoi et al., 2023**

**TABLE OF CONTENTS**

[**Appendix A. Search Strategy. 1**](#_8zc4koh6ejj2)

[**Table S1. Descriptions of current insulin delivery devices. 1**](#_xxltyi3zd4u3)

[**Table S2. Additional glycemic outcomes based on CL machines. 2**](#_w8gwzqfjpyo3)

[**Table S3. GRADE assessment. 3**](#_lczefmysyx66)

[**Figure S1.**](#_c7y1doa9pel7) **Forest plots for (A) DKA and (B) severe hypoglycaemia** [**4**](#_c7y1doa9pel7)

[**Figure S2. Subgroup analysis based on closed-loop system devices for the outcomes of (A) CV and (B) nocturnal hypoglycemia. 5**](#_y7ommcjhcx7)

[**Figure S3. Critical appraisal according to the Cochrane Collaboration’s tool for assessing risk of bias in randomised trials for clinical outcomes.**](#_atrmb3d7f0lk) **7**

[**Figure S4. Critical appraisal according to the Cochrane Collaboration’s tool for assessing risk of bias in randomised trials for functional outcomes.**](#_sof8cffg5k4d) **8**

[**Figure S5. Funnel plots for (A) TIR 70-180 mg/dL and (B) HbA1c shows no evidence of publication bias 9**](#_huycyw9d2b0v)

[**Figure S6. Egger’s regression test does not suggest significant publication bias for (A) HbA1c (%) endpoint**](#_cy2ahjq4lq3)**; but suggests significant publication bias for**  [**(B) % TIR 70-180 mg/dL endpoint. 10**](#_cy2ahjq4lq3)

[**Figure S7. Leave-one-out sensitivity analysis for the outcome of HbA1c (%). 12**](#_83psqvdtpgy6)

[**Figure S8. Baujat plot for the outcome of HbA1c (%). 13**](#_6f9uqoxgaop6)

[**Figure S9. Meta-regression exploring the association between mean differences of HbA1c level (%) and duration of follow-up (weeks). 14**](#_ejtl1ahu7lry)

[**Figure S10. Meta-regression exploring the association between mean differences of HbA1c level (%) and baseline HbA1c (%). 15**](#_dmeotfiejg3j)

# Appendix A. Search Strategy.

*PubMed, Embase and Cochrane:*

(“Type 1 Diabetes” OR “Diabetes Mellitus Type 1” OR T1DM) AND ("Closed Loop" OR “closed-loop” OR "Artificial Pancreas" OR "Bionic Pancreas" OR "Insulin Infusion System" OR "Implantable Programmable Insulin Pump" OR "Artificial Beta Cell" OR “Automated Insulin Delivery” OR AID) AND (RCT OR randomized OR randomised OR random OR randomly)

*Clinicaltrials.gov:*

(“Type 1 Diabetes” OR “Diabetes Mellitus Type 1” OR T1DM) AND ("Closed Loop" OR “closed-loop” OR "Artificial Pancreas" OR "Bionic Pancreas" OR "Insulin Infusion System" OR "Implantable Programmable Insulin Pump" OR "Artificial Beta Cell")

# Table S1. Descriptions of current insulin delivery devices.

| **Name** | **Components** | **Definition** | **Brands/Machines** |
| --- | --- | --- | --- |
| SAP | CGM + insulin pump | Consists of a CGM and an insulin pump that function independently. The insulin pump does not auto-adjust according to the CGM | Omnipod  Medtronic 630G |
| LGTS | CGM + insulin pump + LGTS feature | Programmed insulin pumps that are connected to the CGM and have a feature that suspends insulin release when glucose achieves a certain threshold | MiniMed Paradigm Veo (Medtronic) |
| PLGS | CGM + insulin pump + PLGS | Programmed insulin pumps that are connected to the CGM and have a feature that suspends insulin release when there is a trend in CGM that predicts hypoglycemia in the near future | MiniMed 640G (Medtronic)  t:slim X2 Basal-IQ (Tandem) |
| HCL | CGM + insulin pump + automated algorithm of insulin infusion and suspension | Programmed insulin pumps that are connected to the CGM and have an automated algorithm that can infuse microdoses of insulin or suspend the infusion according to CGM reads and trends. Patients still have to administer bolus insulin on meals based, usually, on insulin-to-carbohydrate ratio | MiniMed 670G (Medtronic)  MiniMed 780G (Medtronic)  t:slim X2 with Control-IQ (Tandem)  CamAPS FX (CamDiab)  DBLG1 (Dbl-diabetes) |
| FCL | CGM + insulin pump + automated algorithm of insulin infusion and suspension + algorithm for bolus infusion | Programmed insulin pumps that are connected to the CGM and have an automated algorithm that can infuse microdoses of insulin or suspend the infusion according to CGM reads and trends. The system can infuse insulin bolus based on qualitative description of the patient’s meals. There is no need for insulin-to-carbohydrate ratio calculation | STG-55 (Nikkiso)  iLet Bionic Pancreas (Beta Bionics) |
| Do-it- yourself AID | CGM + insulin pump + open source algorithms (applications on smartphones) ± communication devices | Insulin pumps and CGM with or without communication devices that allow the user to connect them to open source applications, usually on their smartphones | OpenAPS  AndroidAPS  Loop |
| Bionic Pancreas | CGM + insulin and glucagon pump + automated algorithm for drug delivery and suspension | Insulin and glucagon pump that are connected to the CGM and have an automated algorithm that can infuse both insulin and glucagon | No FDA approved machine |

*AID* Automated Insulin Delivery*, CGM* Continuous Glucose Monitoring, *FCL* Full Closed Loop, *HCL* Hybrid Closed-loop, *LGTS* Low Glucose Threshold Suspend, *PGLS* Predictive Low-glucose Suspend System, *SAP* Sensor-augmented Insulin Pump.

# Table S2. Additional glycemic outcomes based on CL machines.

| **Outcome** | **No of patients (No of comparisons)** | **Pooled Result (CI 95%)** | **P value** | **Heterogeneity** |
| --- | --- | --- | --- | --- |
| **DKA**ª CamAPS FX  t:slim X2 with Control IQ  MiniMed 670G  MiniMed 780G  DBGL1  iLet Bionic Pancreas  OpenAPS  Florence | 450 (4)  607 (5)  635 (5)  119 (2)  126 (1)  225 (1)  165 (3)  86 (1) | 4·02 (0·45 to 36·36)  1·95 (0·30 to 12·53)  0.73 (0.16 to 3.34)  NA  NA  NA  NA  2·67 (0·11 to 67·40) | p = 0·22  p = 0·48  p = 0·69  NA  NA  NA  NA  p = 0·55 | 0%  0%  0%  NA  NA  NA  NA  NA |
| **Severe Hypoglycemia**ª CamAPS FX  t:slim X2 with Control IQ  MiniMed 670G  MiniMed 780G  DBGL1  iLet Bionic Pancreas  OpenAPS  Florence | 489 (4)  552 (5)  593 (4)  119 (2)  126 (1)  NA  185 (3)  200 (3) | 0·74 (0·05 to 11·15)  2·05 (0·75 to 5·59)  0·90 (0·29 to 2·79)  NA  1·72 (0·39 to 7·54)  NA  0·32 (0·01 to 8·23)  4·13 (0·44 to 38·55) | p = 0·83  p = 0·16  p = 0·86  NA  p = 0·47  NA  p = 0·49  p = 0·21 | 33%  0%  23%  NA  NA  NA  NA  0% |
| **% Time (< 54 mg/dl)ᵇ** CamAPS FX  t:slim X2 with Control IQ  MiniMed 670G  MiniMed 780G  DBGL1  iLet Bionic Pancreas  OpenAPS  Florence | 475 (4)  530 ()5  291 (3)  111 (2)  126 (1)  219 (1)  165 (3)  NA | -0·05 (-0·10 to 0·01)  0·03 (-0·04 to 0·10)  -0·45 (-0·87 to -0·02)  -0·46 (-1·29 to 0·37)  -0·50 (-0·70 to -0·30)  -0·03 (-0·10 to 0·04)  -0·23 (-0·42 to -0·05)  NA | p = 0·10  p = 0·46  p = 0·04  p = 0·28  p < 0·001  p = 0·35  p = 0·01  NA | 26%  0%  91%  81%  NA  NA  0%  NA |
| **% Time (< 70 mg/dl)ᵇ**  CamAPS FX  t:slim X2 with Control IQ  MiniMed 670G  MiniMed 780G  DBGL1  iLet Bionic Pancreas  OpenAPS  Florence | 489 (4)  445 (5)  635 (5)  111 (2)  126 (1)  219 (1)  165 (2)  200 (3) | -0·33 (-0·75 to 0·10)  -0·28 (-1·19 to 0·55)  -1·54 (-2·68 to -0·39)  -1·90 (-6·31 to 2·50)  -2·40 (-3·05 to -1·75)  -0·28 (-0·64 to 0·08)  -0·82 (-1·51 to -0·14)  0·42 (-0·09 to 0·94) | p = 0·13  p = 0·51  p = 0·008  p = 0·40  p < 0·001  p = 0·13  p =0·02  p = 0·11 | 85%  83%  92%  88%  NA  NA  28%  92% |
| **% Time (>250 mg/dl)ᵇ** CamAPS FX  t:slim X2 with Control IQ  MiniMed 670G  MiniMed 780G  DBGL1  iLet Bionic Pancreas  OpenAPS  Florence | 232 (2)  395 (3)  333 (4)  111 (2)  126 (1)  219 (1)  129 (2)  NA | -5·46 (-6·95 to -3·97)  -3·14 (-5·44 to -0·83)  -1·63 (-3·06 to -0·20)  -10·55 (-22·70 to 1·60)  -4.30 (-6·20 to -2·40)  -7·62 (-9·57 to -5·67)  -4·12 (-9·21 to 0·97)  NA | p < 0·001  p =0·008  p = 0·03  p = 0·09  p < 0·001  p < 0·001  p = 0·11  NA | 0%  75%  90%  96%  NA  NA  80%  NA |

ª Odds Ratio; ᵇ Mean Difference;

#

# Table S3. GRADE assessment.

|  | **Certainty assessment** | | | | | **Patients** | | **Effect** | **Certainty** |
| --- | --- | --- | --- | --- | --- | --- | --- | --- | --- |
| **Outcome** | **№ of studies** | **Risk of bias** | **Inconsistency** | **Indirectness** | **Imprecision** | **CL** | **UC** | **Absolute**  **(95% CI)** | **Grading** |
| Change in HbA1c (mixed) | 5 | serious^a^ | serious^b^ | not serious | not serious | 514 | 372 | MD **0**·**43 mmol/mol lower**  (0·56 lower to 0·30 lower) | ⨁⨁⨁◯  Moderate |
| Change in HbA1c (adults) | 9 | very serious^c^ | serious^d^ | not serious | not serious | 335 | 332 | MD **0**·**38 mmol/mol lower**  (0·63 lower to 0·12 lower) | ⨁⨁◯◯  Low |
| Change in HbA1c (children and adolescents) | 7 | very serious^c^ | not serious | not serious | not serious | 371 | 286 | MD **0**·**31 mmol/mol lower**  (0·44 lower to 0·19 lower) | ⨁⨁⨁◯  Moderate |
| TIR 70/180 mg/dL (mixed) | 5 | very serious^c^ | serious^e^ | not serious | not serious | 514 | 372 | MD **11**·**21 hours more**  (9·39 more to 13·03 more) | ⨁⨁◯◯  Low |
| TIR 70/180 mg/dL (adults) | 11 | very serious^f^ | serious^g^ | not serious | not serious | 453 | 454 | MD **11**·**69 hours more**  (8·65 more to 14·72 more) | ⨁⨁◯◯  Low |
| TIR 70/180 mg/dL (children and adolescents) | 8 | very serious^c^ | not serious | not serious | not serious | 396 | 310 | MD **9**·**97 hours more**  (8·36 more to 11·58 more) | ⨁⨁⨁◯  Moderate |
| Coefficient of variation (CV) | 23 | very serious^c^ | serious^h^ | not serious | not serious | 1212 | 985 | MD **1**·**09 % lower**  (1·80 lower to 0·39 lower) | ⨁⨁◯◯  Low |
| Nocturnal hypoglycemia | 17 | very serious^c^ | serious^i^ | not serious | not serious | 897 | 764 | MD **1**·**28 mg/dL lower**  (1·76 lower to 0·79 lower) | ⨁⨁◯◯  Low |

# *CI* confidence interval, *MD* mean difference

# a. One study was considered to carry a moderate risk of bias. Downgraded by one level for risk of bias.

# b. Substantially large heterogeneity (I^2^=56%). Downgraded by one level for inconsistency.

# c. More than one study was considered to carry a high risk of bias. Downgraded by two levels for risk of bias.

# d. Considerable large heterogeneity (I^2^=88%). Downgraded by one level for inconsistency.

# e. Substantially large heterogeneity (I^2^=51%). Downgraded by one level for inconsistency.

# f. More than one study was considered to carry a moderate risk of bias. Downgraded by two levels for risk of bias.

# g. Considerable large heterogeneity (I^2^=89%). Downgraded by one level for inconsistency.

# h. Considerable large heterogeneity (I^2^=81%). Downgraded by one level for inconsistency.

# i. Considerable large heterogeneity (I^2^=84%). Downgraded by one level for inconsistency.

#

# Figure S1. Forest plots for (A) DKA and (B) severe hypoglycaemia
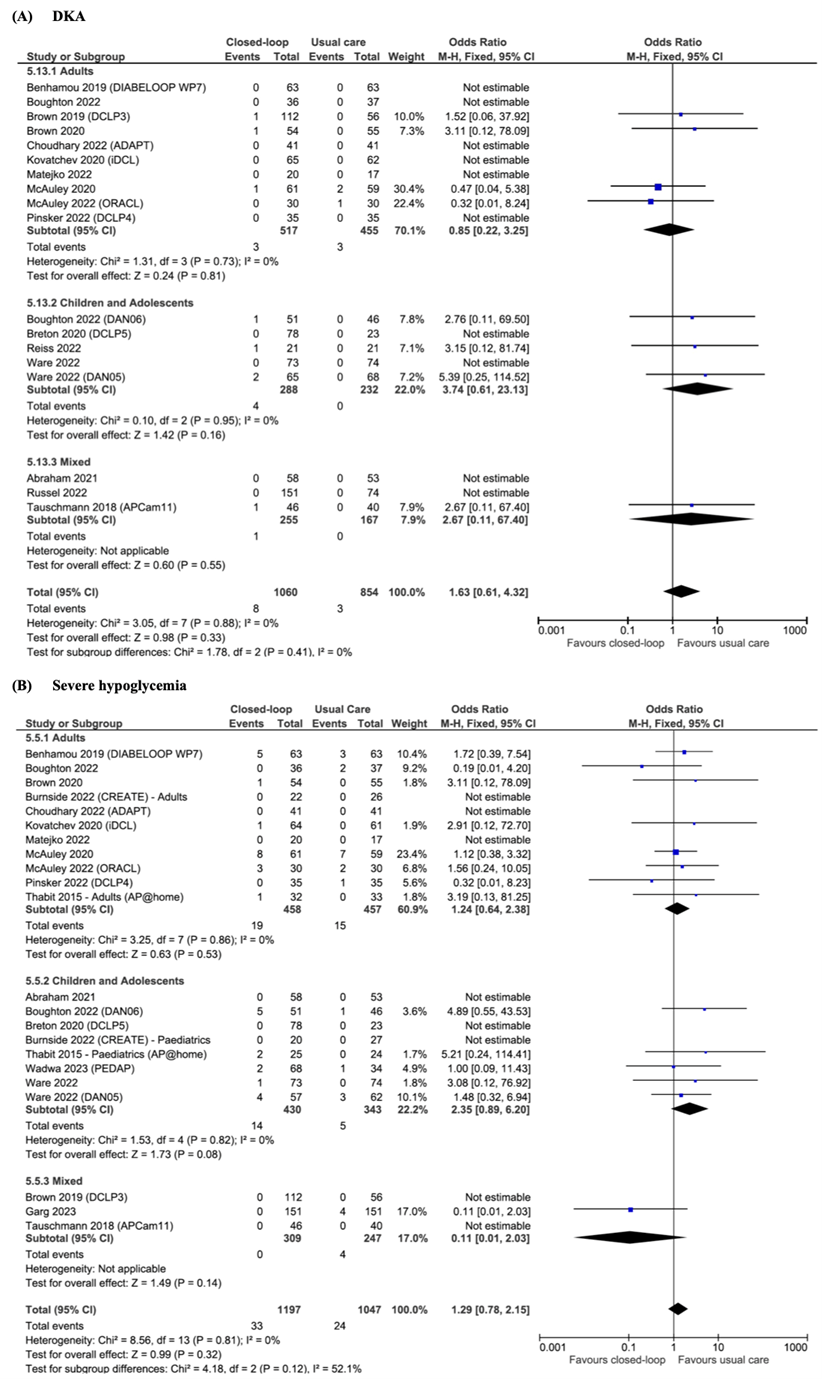


# Figure S2. Subgroup analysis based on closed-loop system devices for the outcomes of (A) CV and (B) nocturnal hypoglycemia.

**A) CV**

**
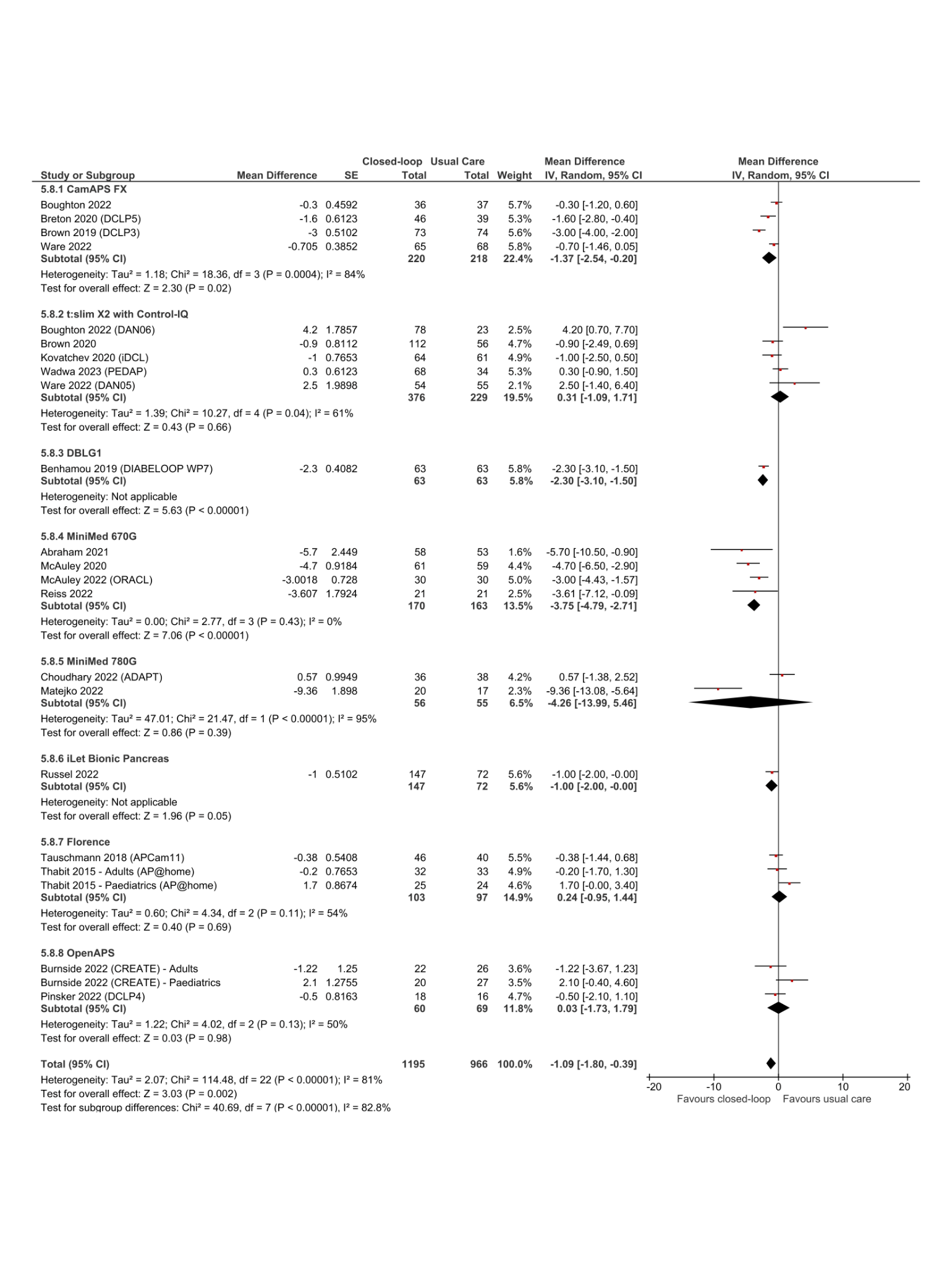
**

**B) Nocturnal hypoglycemia**

**
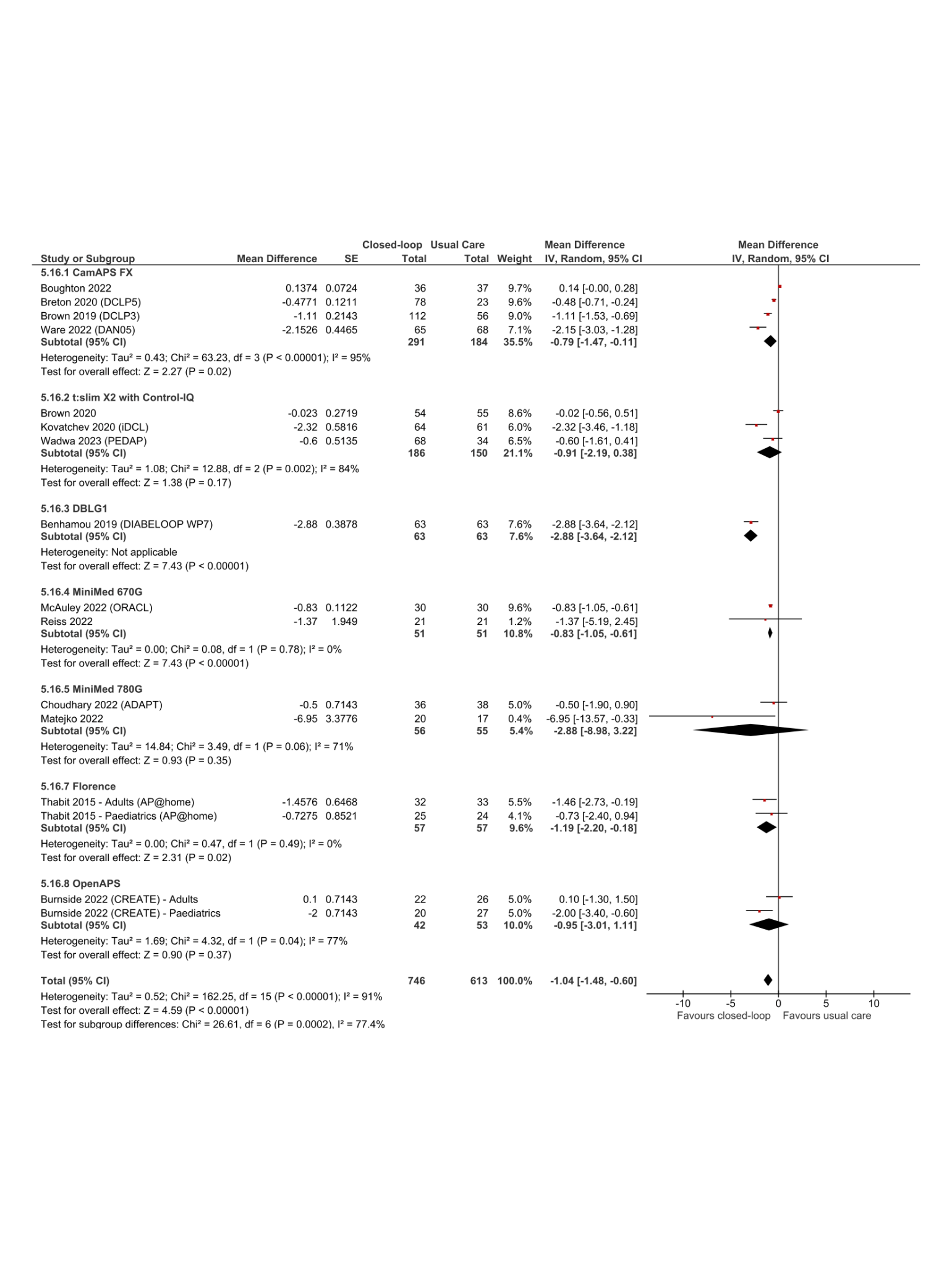
**

# Figure S3. Critical appraisal according to the Cochrane Collaboration’s tool for assessing risk of bias in randomised trials for clinical outcomes.


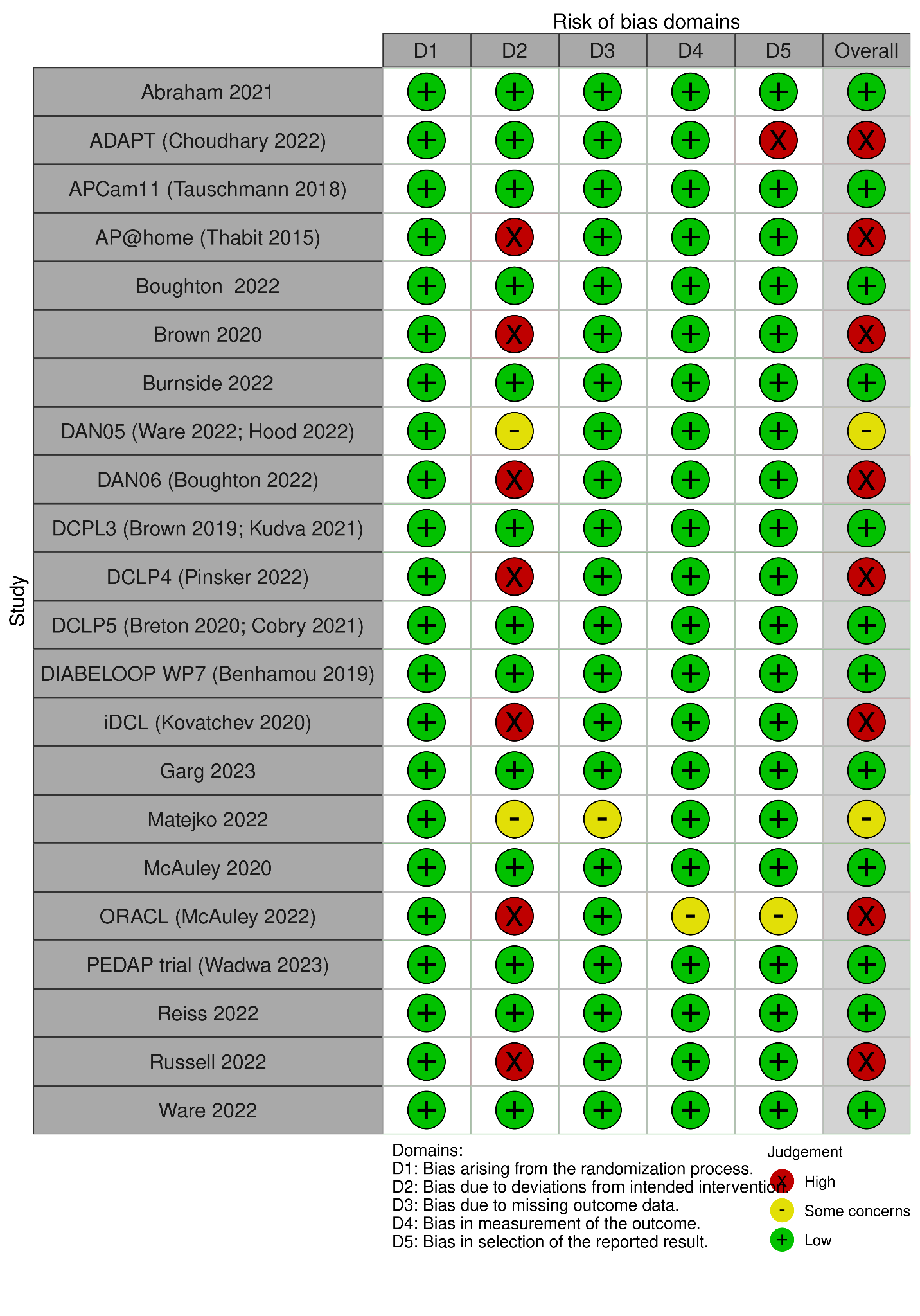


# Figure S4. Critical appraisal according to the Cochrane Collaboration’s tool for assessing risk of bias in randomised trials for functional outcomes.


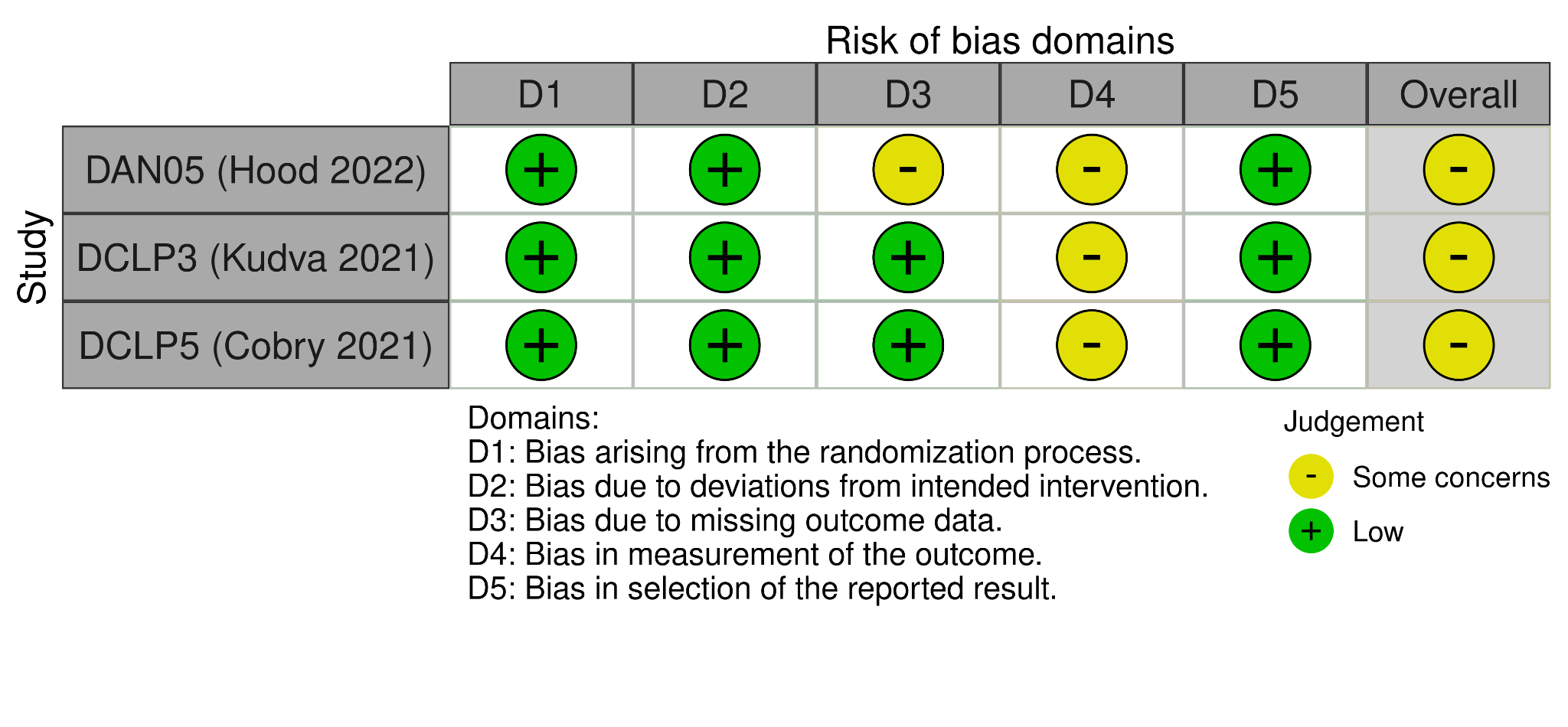


#

# Figure S5. Funnel plots for (A) HbA1c % and (B) TIR 70-180 mg/dL show no evidence of publication bias.


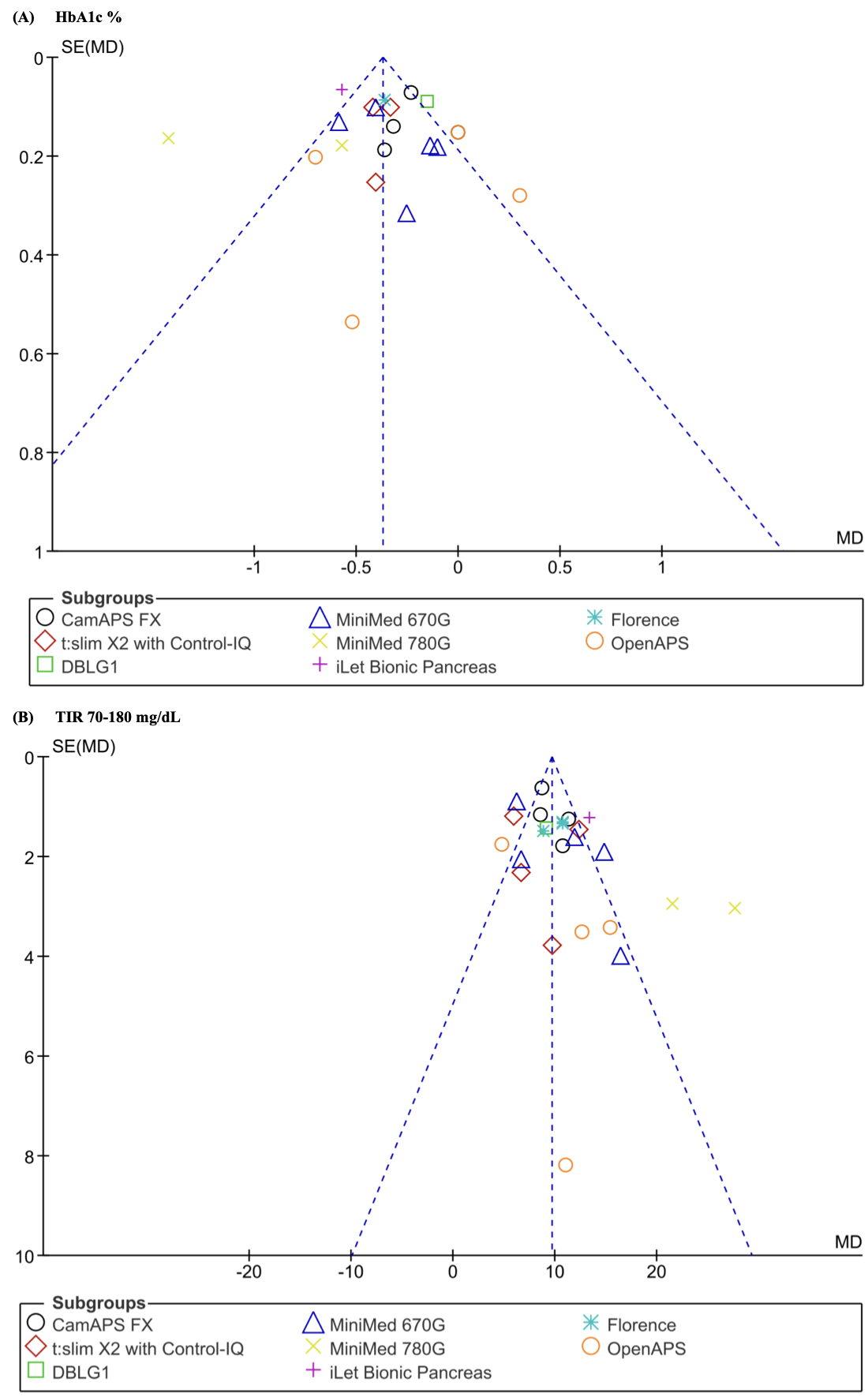


# Figure S6. Egger’s regression test does not suggest significant publication bias for (A) HbA1c (%) endpoint; but suggests significant publication bias for (B) % TIR 70-180 mg/dL endpoint.

**A) HbA1c**


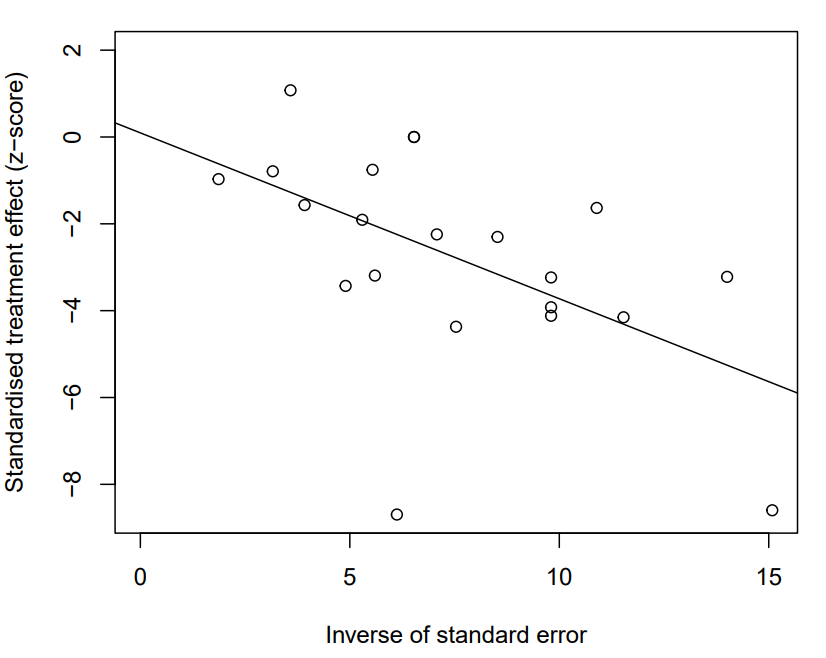


|  | **t** | **df** | **p-value** |
| --- | --- | --- | --- |
| **Egger’s test** | 0·08 | 19 | 0·9333 |

**B) % TIR 70-180 mg/dL**

**
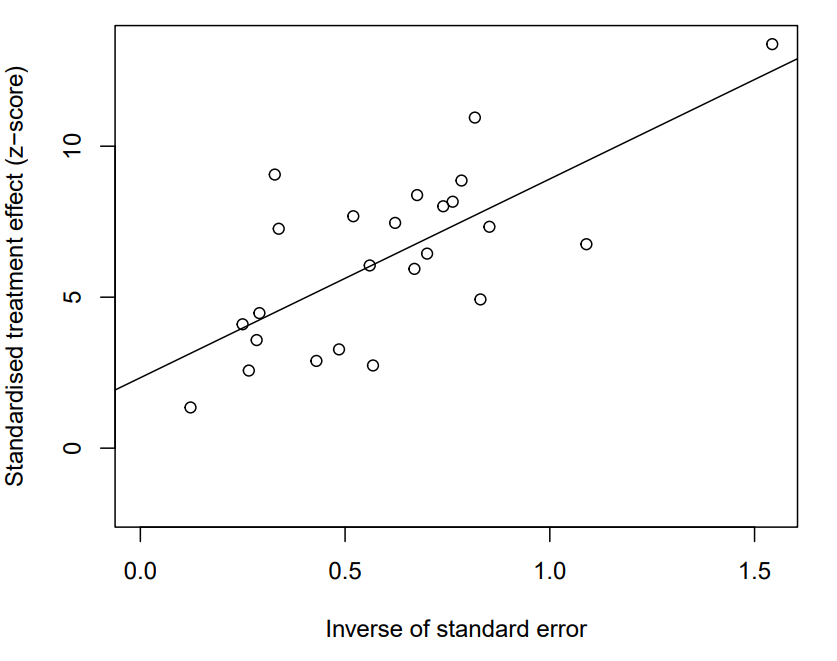
**

|  | **t** | **df** | **p-value** |
| --- | --- | --- | --- |
| **Egger’s test** | 2·49 | 22 | 0·0209 |

# Figure S7. Leave-one-out sensitivity analysis for the outcome of HbA1c (%).


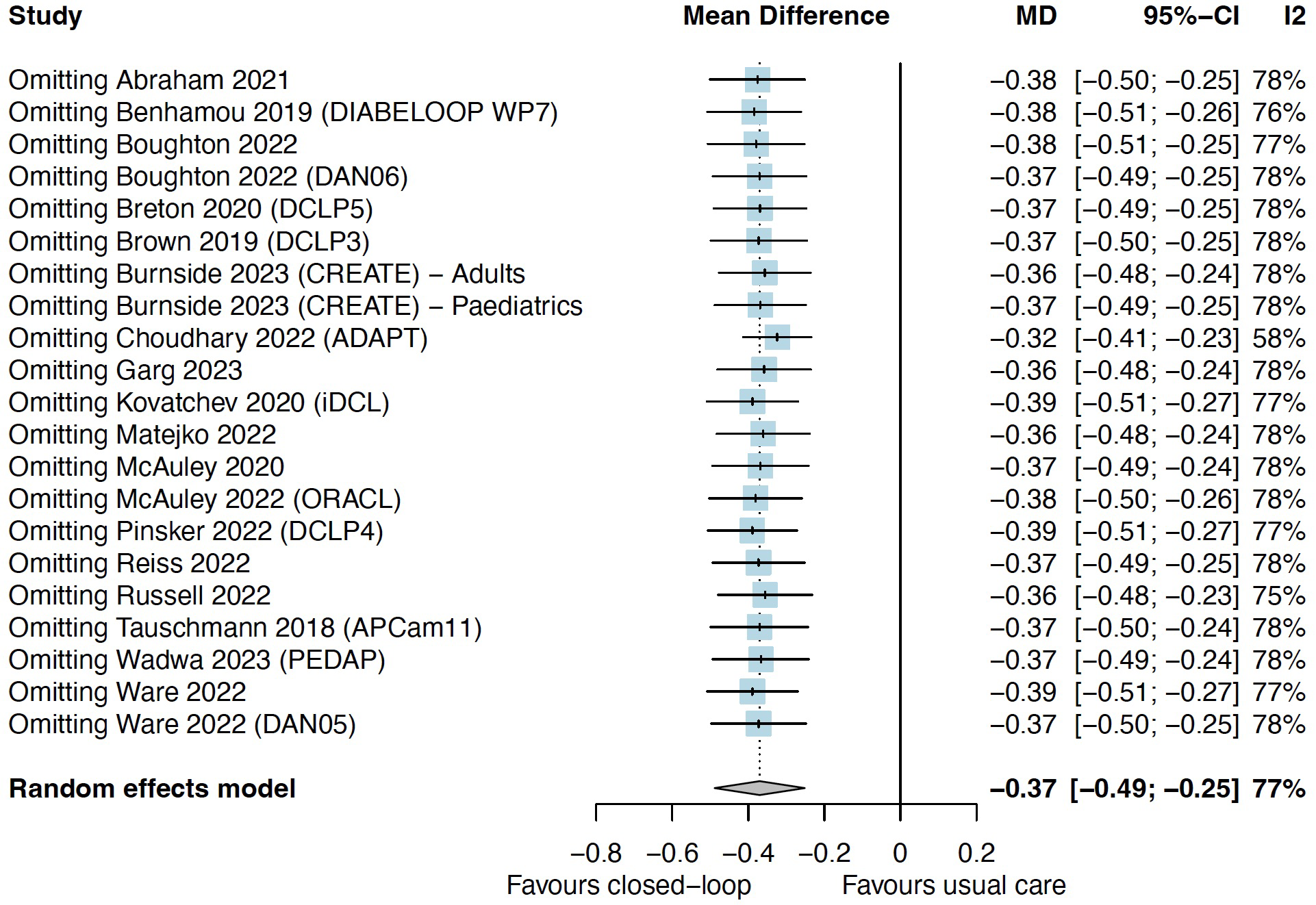


#

# Figure S8. Baujat plot for the outcome of HbA1c (%).


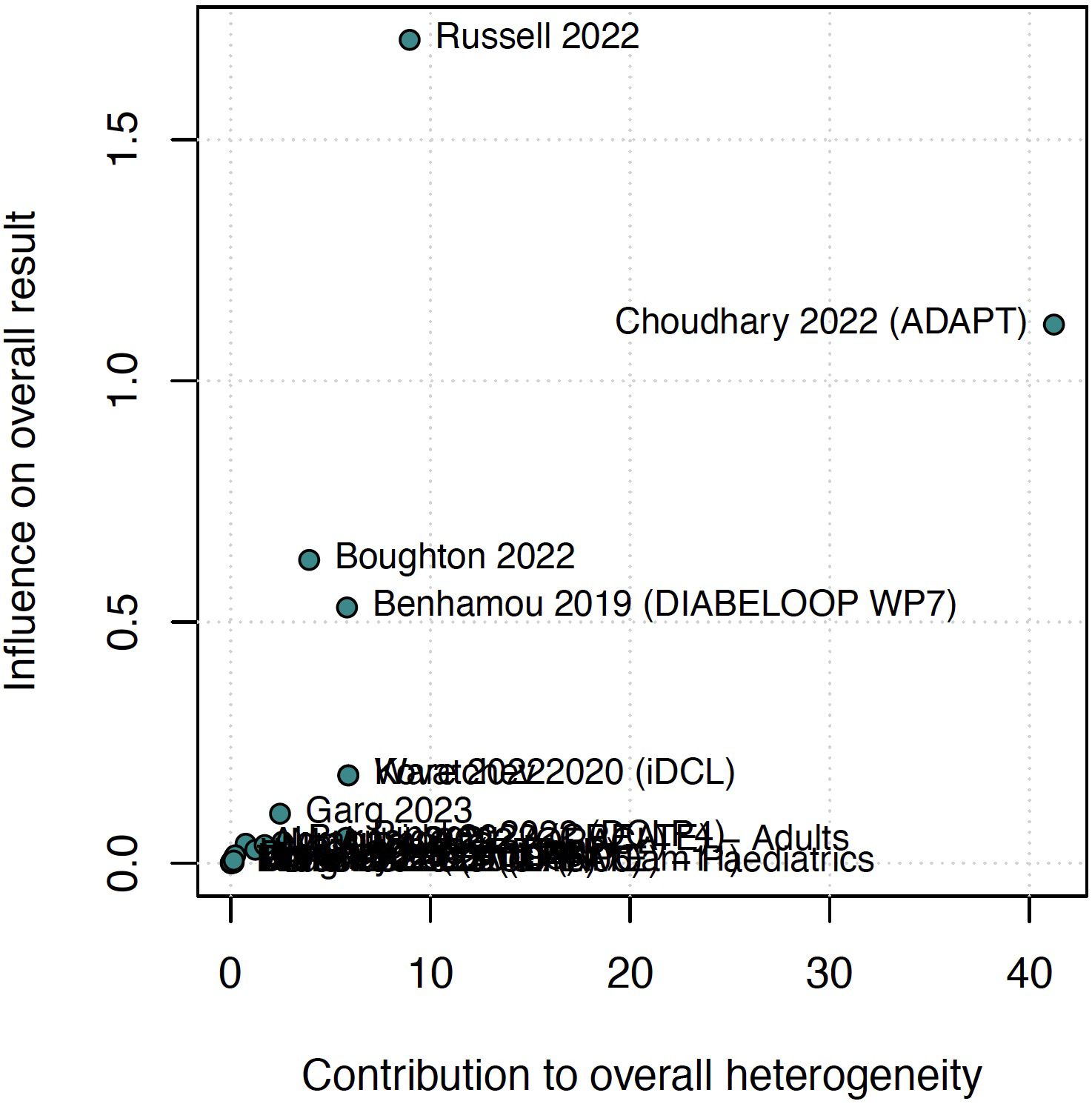


# Figure S9. Meta-regression exploring the association between mean differences of HbA1c level (%) and duration of follow-up (weeks).


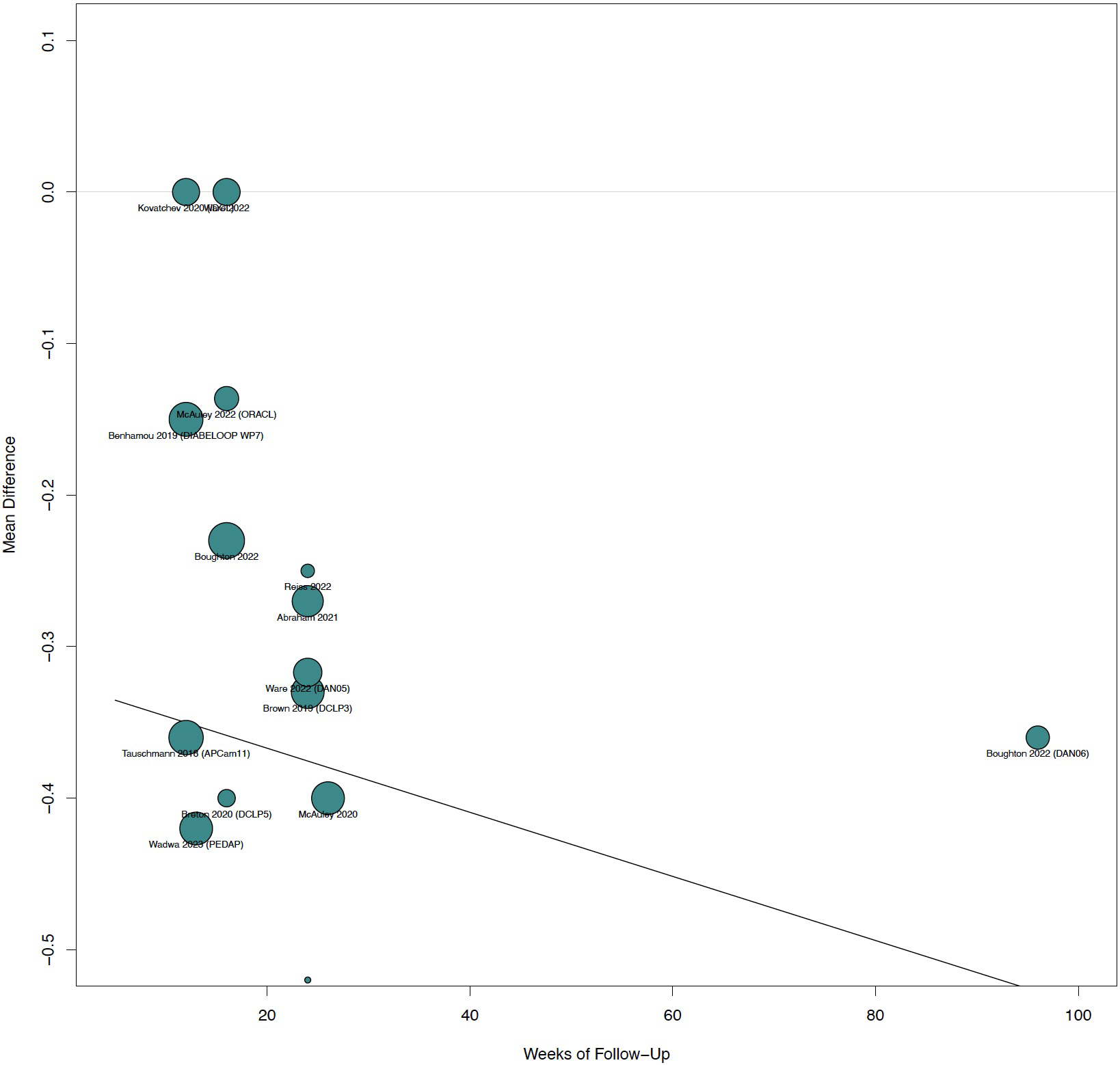


|  | Effect Estimate | p-value | $I^{2}$ | Test for Residual Heterogeneity |
| --- | --- | --- | --- | --- |
| Intercept | -0·3247 | 0·0012 | 78·02% | P < 0·001 |
| DM2 | -0·0021 | 0·5688 |  |  |

# Figure S10. Meta-regression exploring the association between mean differences of HbA1c level (%) and baseline HbA1c (%).


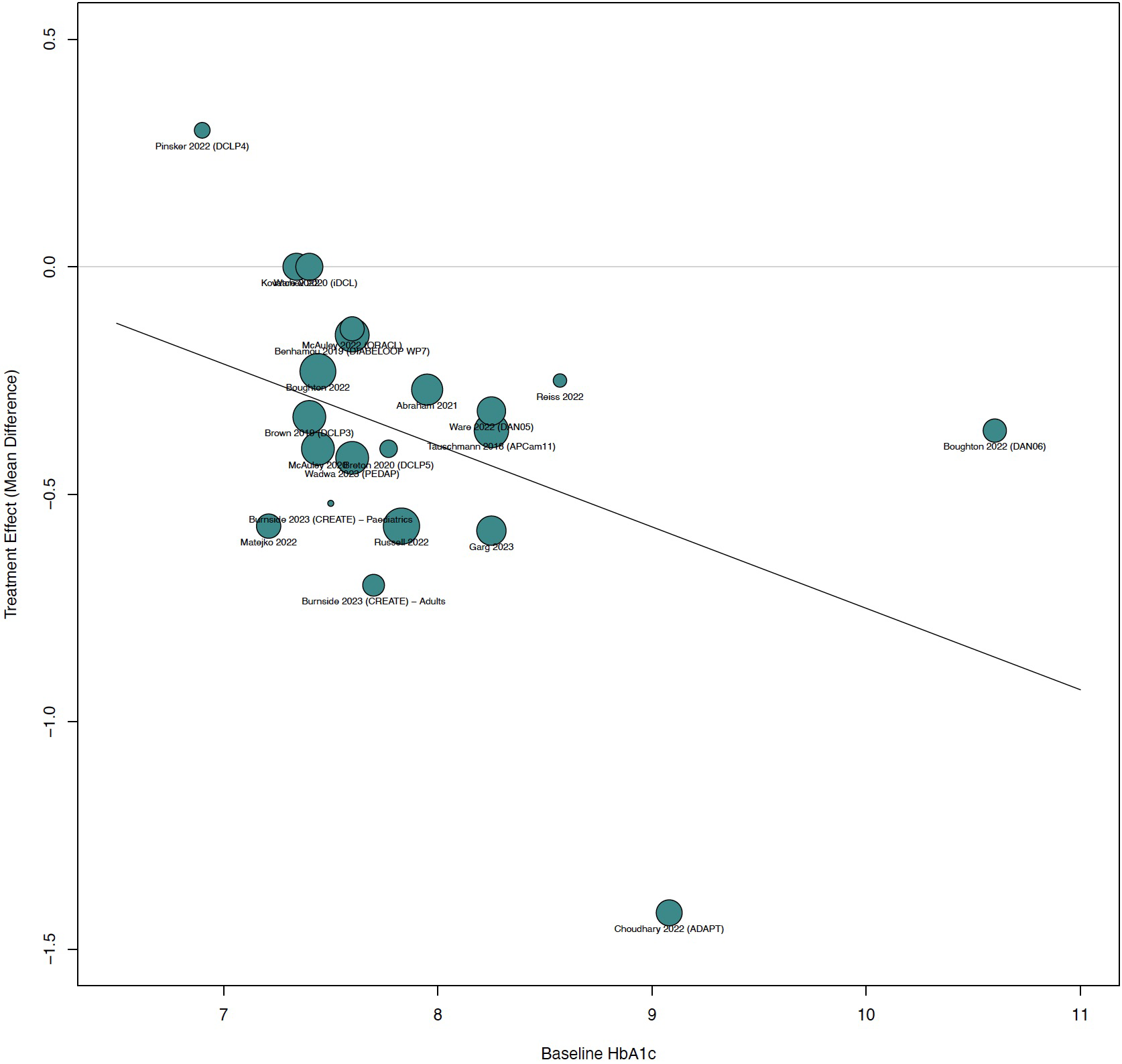


|  | Effect Estimate | p-value | $I^{2}$ | Test for Residual Heterogeneity |
| --- | --- | --- | --- | --- |
| Intercept | 1·0396 | 0·0883 | 73·07% | P < 0·001 |
| DM2 | -0·1790 | 0·0203 |  |  |
